# Single-Cell RNA Sequencing Analysis Reveals the Role of Macrophage-Mediated CD44–AKT–CCL2 Pathways in Renal Tubule Injury during Calcium Oxalate Crystal Formation

**DOI:** 10.34133/research.0690

**Published:** 2025-05-06

**Authors:** Xi Jin, Zhongyu Jian, Yucheng Ma, Jun Wen, Ningning Chao, Xiaoting Chen, Liyuan Xiang, Yiqiong Yuan, Linhu Liu, Ya Li, Jingwen Wei, Banghua Liao, Li Zhang, Kunjie Wang

**Affiliations:** ^1^ Department of Urology, Institute of Urology (Laboratory of Reconstructive Urology), West China Hospital, Sichuan University, Chengdu, Sichuan, P.R. China.; ^2^ Department of Pulmonary and Critical Care Medicine, Institute of Respiratory Health, State Key Laboratory of Respiratory Health and Multimorbidity, Frontiers Science Center for Disease-related Molecular Network, Sichuan Provincial Engineering Laboratory of Precision Medicine, Precision Medicine Key Laboratory of Sichuan Province, West China Hospital, West China School of Medicine, Sichuan University, Chengdu 610041, Sichuan Province, P.R. China.; ^3^ Animal Experimental Center, West China Hospital, Sichuan University, Chengdu, Sichuan, P.R. China.

## Abstract

Oxalate-induced crystalline kidney injury is a common form of crystal nephropathy. The accumulation of calcium oxalate (CaOx) crystal could lead to renal epithelium injury and inflammation. The underlying cellular events in kidney after CaOx crystal formation are largely unknown. This study was aimed to gain a better understanding of mouse kidney function in the development of renal CaOx formation. The study utilized a mouse CaOx model to analyze the cellular response at 5 time points using single-cell RNA sequencing and investigate the interaction of different cells during renal CaOx crystal formation. Additionally, the study investigated the communication between these cells and macrophages, as well as the role of chemokines in recruiting infiltrating macrophages. RNA velocity analysis uncovered an alternative differentiation pathway for injured and S1 proximal tubule cells, which mainly communicate with macrophages through the SPP1–CD44 pair, along with the expression of proinflammatory factors and stone matrix genes during renal CaOx crystal formation. Furthermore, resident Fn1 macrophages were found to express chemokines, such as CCL2, which recruited infiltrating macrophages. The CCL2 secretion was mediated by the CD44–AKT pathway. Blocking CCL2 decreased the expression of injury markers in the kidney, including CLU, LCN2, and KIM-1, and inhibited CaOx crystal deposition. The study identified potential cell types and target genes involved in renal tubule injury in oxalate-related crystal nephropathy. The findings shed light on the cellular processes that contribute to the formation and damage caused by CaOx crystals within the kidney, which could lead to the development of potential cell types and target genes for treating this condition.

## Introduction

The kidney is the organ that is particularly vulnerable to crystal formation, which arises because of the mineral secretion and the urine supersaturation concentration [1]. Over time, the accumulation of crystals can result in the emergence of diverse forms of crystal nephropathies [1]. Furthermore, these conditions can progress to more severe forms of kidney stone diseases, where the crystals coalesce to form larger, more problematic stones that can block the urinary tract and cause considerable pain and other complications [2,3]. Oxalate-induced crystalline kidney injury is a prevalent type of crystal nephropathy [4]. Primary hyperoxaluria and secondary hyperoxaluria can contribute to the formation of calcium oxalate (CaOx) crystals and stone in kidney [5,6]. CaOx stones are a prevalent variety of kidney stones, with their incidence varying from continent to continent [7,8]. The prevalence of kidney stones is roughly between 7% and 13% in North America, 5% and 9% in Europe, and 1% and 5% in Asia [9–11]. However, with the lifestyle and dietary habit changes that are occurring nowadays, the incidence of CaOx kidney stones is gradually increasing worldwide, resulting in a heavy financial burden [12–14].

The creation of CaOx stones involves a multifaceted process, including an initial nucleation step, followed by crystal growth and aggregation within the renal tubules [15–18]. Many studies have indicated that renal tissue is susceptible to injury and inflammation from high levels of oxalate exposure or CaOx crystal, making it more prone to senescence [19–22]. Several studies have investigated oxalate-induced kidney injury, encompassing inflammatory responses and oxidative stress [23,24]. Despite these experimental efforts of in vitro or in vivo, the molecular mechanisms of oxalate crystal-induced kidney injury need to be fully understood.

For the past few years, the emergence of single-cell RNA sequencing (scRNA-seq) technology has offered new concepts and tools for studying CaOx crystal formation in the kidneys. This study was aimed to explore the molecular mechanism of CaOx crystal-induced injury in mouse kidneys using scRNA-seq technology, providing new perspectives and ideas to better understand and treat oxalate-related crystal nephropathy.

## Results

### Identification of cell populations in mouse kidneys with CaOx crystals

**Fig. 1.**
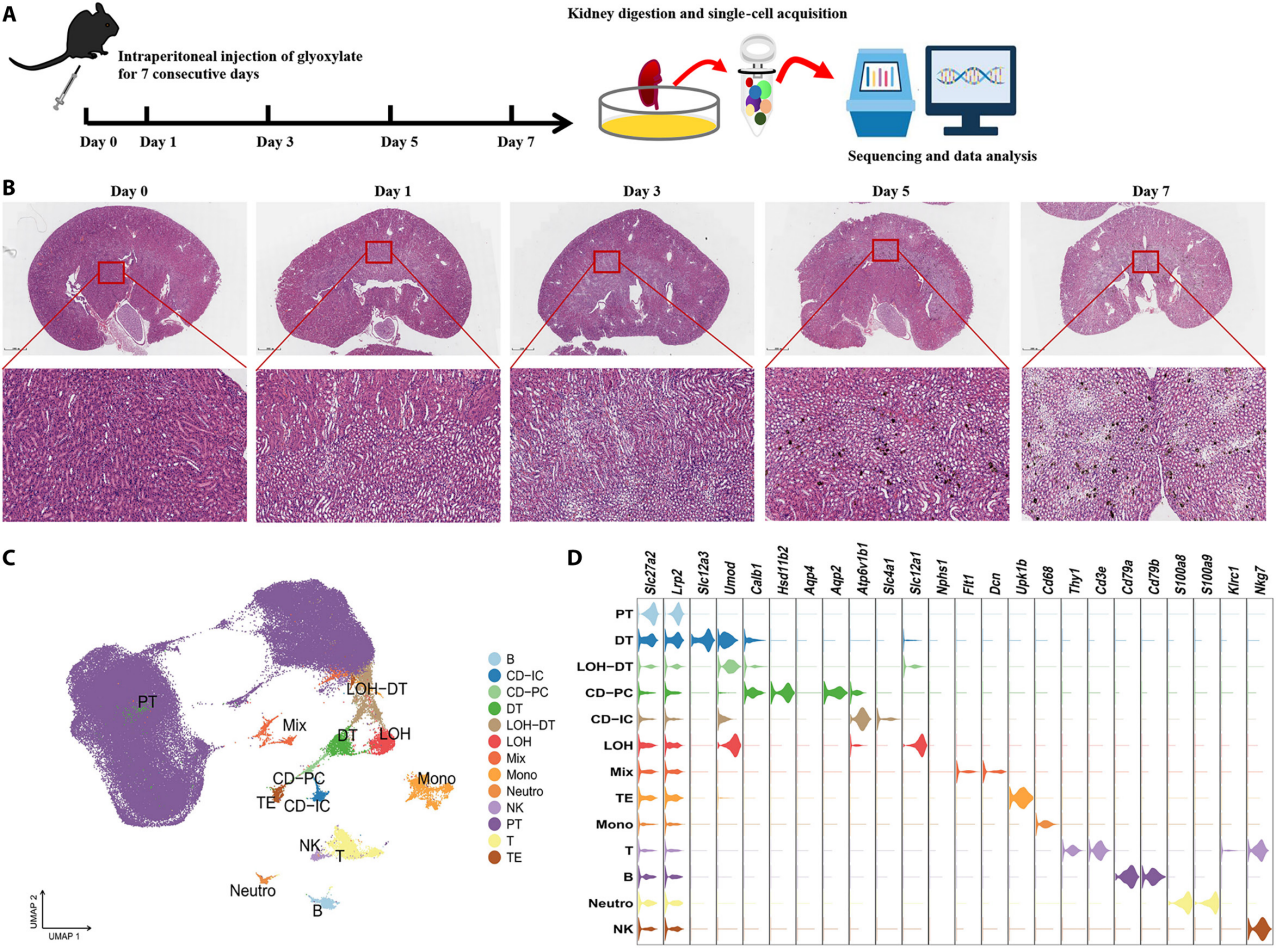
scRNA-seq workflow and UMAP plots. (A) Workflow of scRNA-seq of mouse kidneys. (B) Representative H&E and VK stainings of kidney sections of different days, and CaOx depositions were black or brown dot plots. (C) UMAP plots for the distribution of the 13 major cell populations. B, B cells; CD-IC, collecting duct-intercalated cells; CD-PC, collecting duct principal cells; DT, distal tubules; LOH, loop of Henle; MIX, mix cluster including podocytes, endothelial cells, and fibroblasts; Mono, monocytes; Neutro, neutrophils; NK, NK cells; PT, proximal tubules; T, T cells; TE, transitional epithelium. (D) Violin plots present marker genes for the different cell types.

We intraperitoneally injected mice with glyoxylate, collected their kidneys at different time points (Fig. 1A), and stained the kidney slices using von Kossa (VK) followed by hematoxylin and eosin (H&E) staining (Fig. 1B). The proximal convoluted tubules were mildly swollen on the first day after glyoxylate administration. Three days after glyoxylate administration, a few CaOx crystals were found in the proximal tubules (PTs), and the partial proximal convoluted tubules, collecting ducts, and loops of Henle epithelial cells were swollen. However, on days 5 and 7, more CaOx crystals were found in cortico-medullary junction or the medulla, and few CaOx crystals were found in cortex (Fig. 1B and Fig. S1). Tubule dilation, swelling, slight necrosis, and inflammatory cell infiltration were observed (Fig. 1B and Fig. S1).

scRNA-seq was performed using a CaOx mouse model at multiple CaOx stages. After quality control filtering, we obtained 109,395 cells from the mouse kidneys. Unsupervised clustering was employed to identify the renal cell populations. The UMAP (Uniform Manifold Approximation and Projection) plots revealed the cell clusters, including the PTs, distal tubules (DTs), loops of Henle, collecting duct-intercalated and collecting duct principal cells, transitional epithelium, podocytes, fibroblasts, endothelial cells, monocytes, T cells, B cells, natural killer (NK) cells, and neutrophils (Fig. 1C). The cell clusters were identified and annotated according to the expression patterns of established marker genes, such as *Slc27a2* and *Lrp2* for PTs; *Slc12a3* for the DTs; *Slc12a1* and *Umod* for the loop of Henle (LOH); *Atp6v1b1* and *Slc4a1* for collecting duct-intercalated cells; *Hsd11b2*, *Aqp4*, and *Aqp2* for collecting duct principal cells; and *Upk1b* for the transitional epithelium (Fig. 1D and Table S1). One mix cluster was combined from 2 cell types (LOH-DT), based on their expression of *Slc12a1*, *Umod*, *Calb1*, and *Pvalb* (Fig. 1D and Table S1). Another mix cluster was divided into 3 cell types: podocytes (*Podxl*; Fig. S1A), endothelial cells (*Flt1*; Fig. S2A), and fibroblasts (*Dcn*, *Col1a1*, and *Col3a1*; Fig. S2A). Immune cells were identified by their expression of specific markers: *Thy1* and *Cd3e* for T cells, *Cd79a* and *Cd79b* for B cells, *S100a8* and *S100a9* for neutrophils, and *Klrc1* and *Nkg7* for NK cells (Fig. 1C and Table S1). Monocytes were clustered into a single-cell population, which was identified based on *Cd68* expression (Fig. 1C and Table S1). However, this cell population highly expressed dendritic cell marker genes such as *Clec9a* and *Cd209a* (Fig. S2B).

### Injured PTs promote kidney injury and inflammation during CaOx crystal formation

**Fig. 2.**
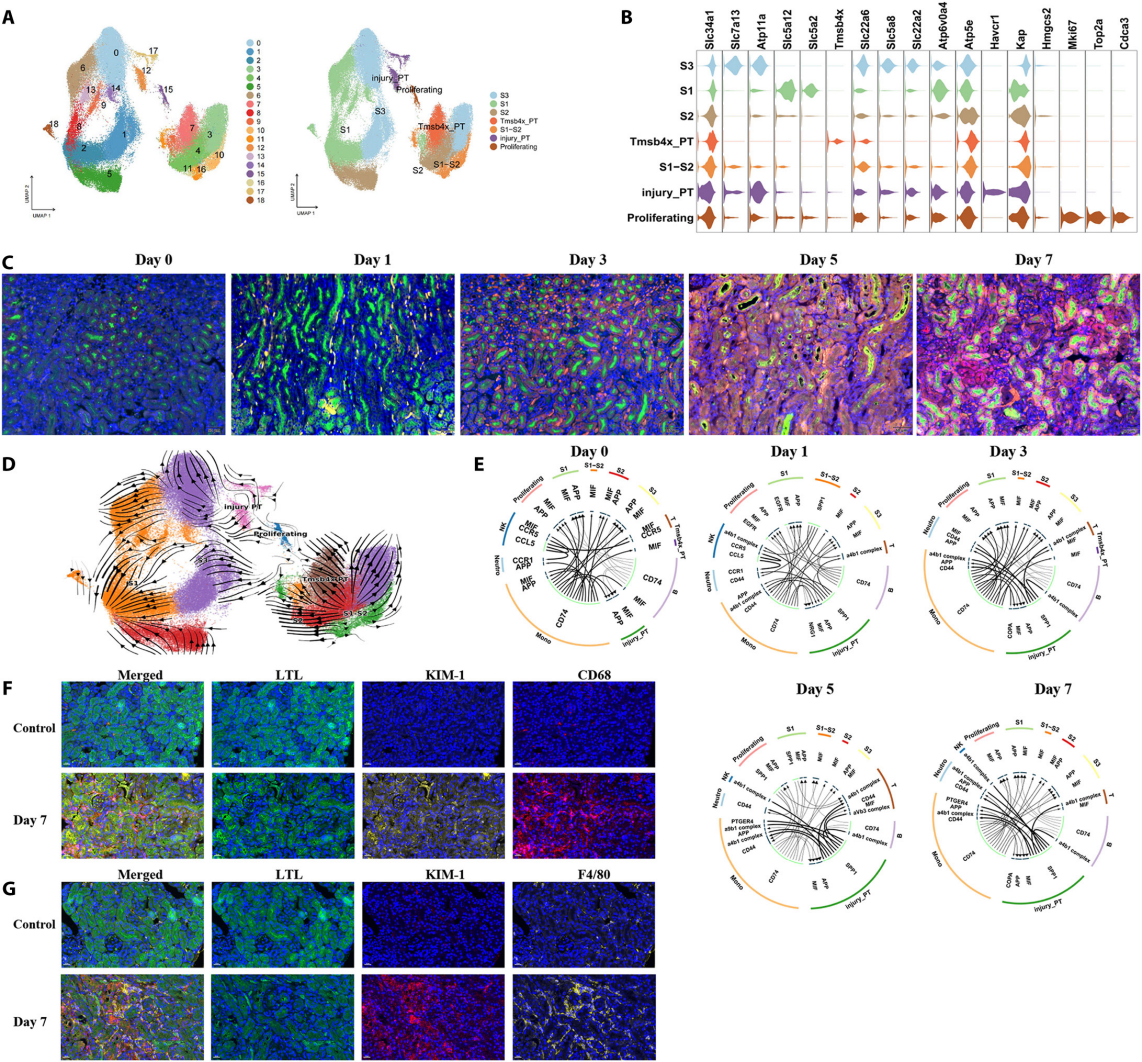
Characterizations of PTs in the kidney. (A) UMAP plots for the distribution of the PT subtypes. (B) Violin plots present marker genes for the different PTs. (C) Representative immunofluorescence stainings of KIM-1 (*Havcr1*, red), LTL (green), and 4′,6-diamidino-2-phenylindole (DAPI) (blue for nuclei) on kidney sections at different days. Scale bar, 50 μm. (D) UMAP plot of PT clusters indicating developmental transition as revealed by RNA velocity. (E) Crosstalk between PTs and immune cells at different days. (F) Representative multiple fluorescent of LTL (green), KIM-1 (yellow), and CD68 (red) in kidney. (G) Representative multiple fluorescent IHC of F4/80 (yellow), KIM-1 (red), and LTL (green) in kidneys. DAPI (blue) for nuclei. Scale bar, 20 μm.

Renal tubular epithelial injury is a critical factor in the formation of CaOx kidney stones and is regarded as a primary trigger for their development [25,26]. First, we studied the characteristics of PT cells during CaOx crystal formation in mouse kidneys. The PT cells were subsequently subclustered into 18 cell types (Fig. 2A and Table S2). Clusters 2, 6, 8, 9, 13, 14, and 18 expressed *Slc5a12* and were defined as S1 PT cells [27,28] (Fig. 2B). Clusters 4 and 5 were defined as S2 PT cells [29,30] since they expressed *Slc34a1*, *Slc13a3*, *Slc22a6*, *Atp5e*, *Hmgcs2*, and *Atp6v0a4* (Fig. 2B). Clusters 0, 1, and 3 expressed *Slc7a13* and *Atp11a* and were defined as S3 PT cells [29,31] (Fig. 2B). Clusters 10, 11, and 16 were defined as S1–S2 PT cells [28,29] and expressed *Slc22a6* (Fig. 2B). The analysis unveiled a subcluster characterized by a high expression of *Mki67* (Fig. 2B), likely representing proliferating PT cells, which was enriched in proteins related to oxidative phosphorylation, according to the Gene Set Enrichment Analysis (GSEA) results (Fig. S3). Notably, another subcluster with a high expression of *Havcr1* (KIM-1), a well-known molecule associated with injury (Fig. 2B) whose expression is increased during CaOx crystal formation in mouse kidneys (Fig. 2C), was identified.

To assess the potential origin and cellular differentiation of the PT clusters, RNA velocity analysis was conducted. We observed a differentiation pathway for injured PT and S1 PT cells in CaOx kidneys that was different from that of S3, S2, or S1–S2 PT cells (Fig. 2D and Table S3). Notably, the subcluster with a high *Havcr1* expression level also highly expressed *Spp1*, *Fgg*, *Fga*, and *Fgb*, which are enriched in cell adhesion (Fig. S4) and are found in the stone matrix [32]. Notably, injured PT cells also expressed proinflammatory and profibrotic genes, including *Cxcl1*, *Tnfrsf12a*, and *Pdgfb*, which play a role in regulating the renal CaOx formation (Fig. S5). The regulation of Toll-like receptors (TLRs) by endogenous factors, TLR cascades, and extracellular matrix organization was highly evident in injured PT cells (Fig. S4). The GSEA results showed that PT injury states were related to the negative regulation of the epithelial cell apoptotic process and heterotypic cell–cell adhesion from the first day of glyoxylate administration. Moreover, these states then started being associated with terms such as positive regulation of interleukin-8 production and regulation of extrinsic apoptotic signaling pathway via death domain receptors during the progression of CaOx formation (Fig. S6). In contrast with what was found in proliferating cells, oxidative phosphorylation was not found in injured PTs during the early stages of CaOx formation (Figs. S3 and S6). Vcam1, as failed repaired PT marker [33], was increased in injured PTs at days 5 and 7 (Fig. S7). These results suggest that these small cell populations failed to repair and induce apoptosis during CaOx formation.

S1 PT cells highly expressed fibroinflammatory genes, such as *Pdgfb*, *Cxcl16*, *C3*, *Nkfb2*, and *Tnfrsf12a* (Fig. S8). On day 1 of glyoxylate administration, the GSEA results showed that the S1 PT cells were enriched in response to interferon-β, type I interferon, and the positive regulation of interferon-β production (Fig. S9). On day 3 of CaOx formation, the GSEA results obtained from the S1 PT cells revealed terms such as “eosinophil chemotaxis” and “migration”. On day 5, the cells were enriched in heterotypic cell–cell adhesion, antimicrobial humoral responses, and granulocyte migration. On day 7, the GSEA results of S1 PT cells revealed terms such as “humoral immune response”, “response to chemokines”, and “granulocyte migration” (Fig. S9). Taken together, these results reveal that S1 PTs contribute to the persistence of inflammatory responses.

Since injured PTs and S1 PTs express proinflammatory cytokines and chemokines, we explored the cell–cell interactions in CaOx kidneys based on the receptor and ligand expressions that communicate with immune cells. In the control group, monocytes interacted with the PTs to maintain homeostasis (Fig. 2E). However, after glyoxylate administration, the injured PTs mainly communicated with monocytes through the SPP1–CD44 complexes (Fig. 2E). As CaOx crystals started forming in the kidneys, the interaction signals between the injured PTs and immune cells were enhanced, while monocytes communicated with other cells through the CD74–APP and CD74–MIF pairs (Fig. 2E). We found that few monocytes were around tubule cells in control mouse kidneys; however, there were a large number of monocytes, especially macrophages, surrounding the injured renal tubules in CaOx kidneys (Fig. 2F and G). These results suggest that injured PTs attract immune cells to injured renal tubule cells, and that activated monocytes interact with other cell clusters to promote injury and inflammation during CaOx crystal formation.

### Fn1-resident macrophages secrete chemokines that recruit infiltrating macrophages via the CCL2 pathway

**Fig. 3.**
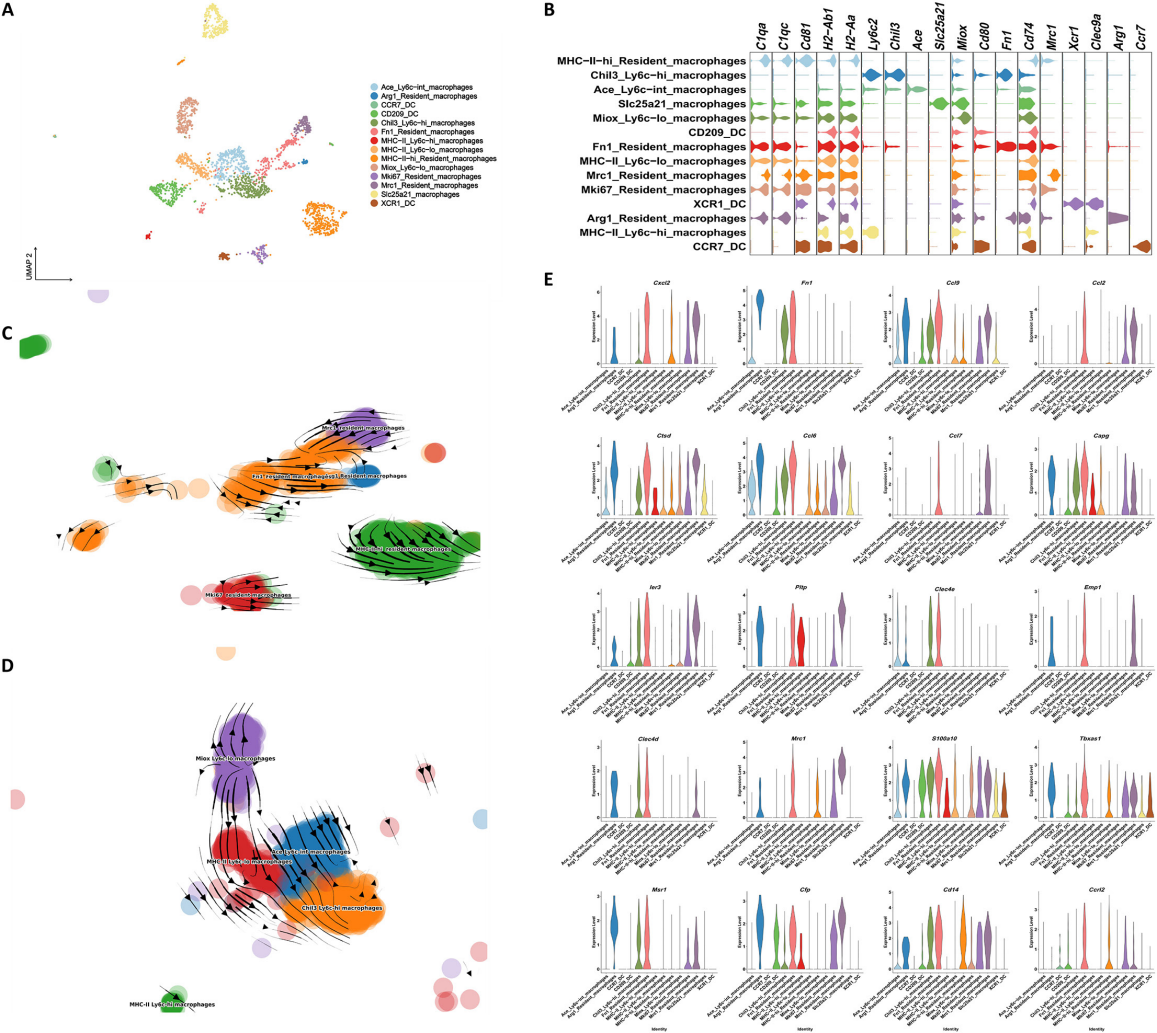
Characterizations of monocytes in the kidney. (A) UMAP plots for the distribution of the monocytes subtypes. (B) Violin plots present marker genes for the different monocytes. (C) UMAP plot of RM clusters indicating developmental transition as revealed by RNA velocity. (D) UMAP plot of infiltrating macrophage clusters indicating developmental transition as revealed by RNA velocity. (E) Violin plots show the expression of many chemokines in Fn1 RMs on day 1.

Owing to the heterogeneity of monocytes, which are the main cells communicating with PTs, LOH, DTs, and collecting duct cells (Fig. 2E and Fig. S10), we recruited monocytes to explore their role in CaOx crystal formation. Monocytes were divided into 14 subgroups, including macrophages and dendritic cells, using unsupervised clustering (Fig. 3A and B and Fig. S11). Five clusters highly expressed typical resident macrophage (RM) marker genes [34,35], such as *C1qa*, *C1qc*, and *Cd81* (Fig. 3B), as well as major histocompatibility complex class II (MHC-II) genes (*Cd74*, *H2-Aa*, and *H2-Ab1*), which may be involved in antigen presentation and immune regulation. RNA velocity analysis revealed an alternative differentiation pathway from that of Fn1 RMs [36,37] and Mki67 RMs [37,38] into MHC-II^hi^ RMs in CaOx kidneys (Fig. 3C and Table S4). Under homeostatic conditions, the Fn1 RMs, Mki67 RMs, and MHC-II^hi^ RMs were mainly enriched in the Gene Ontology (GO) terms “ribosome biogenesis” and “antigen processing and presentation” (Fig. S12A to C). After glyoxylate administration, the enrichment of Fn1 RMs in the GO terms “regulation of leukocyte differentiation”, “antigen processing and presentation of exogenous peptide antigen”, and “MHC-II class protein complex assembly” decreased (Fig. S12D). However, from days 3 to 7 post-hyperoxaluria, the enrichment of the Fn1 RMs in the GO terms “myeloid leukocyte activation”, “phagocytosis”, “leukocyte migration”, and “myeloid leukocyte differentiation” increased (Fig. S12E and F). On days 5 and 7 after glyoxylate administration, the enrichment in the GO terms related to DNA replication and repair decreased in Mki67 RMs (Fig. S12G and H), indicating self-repair failure. On day 7 after CaOx deposition, the differential expression of MHC-II^hi^ RMs was involved in myeloid leukocyte migration (Fig. S12I).

**Fig. 4.**
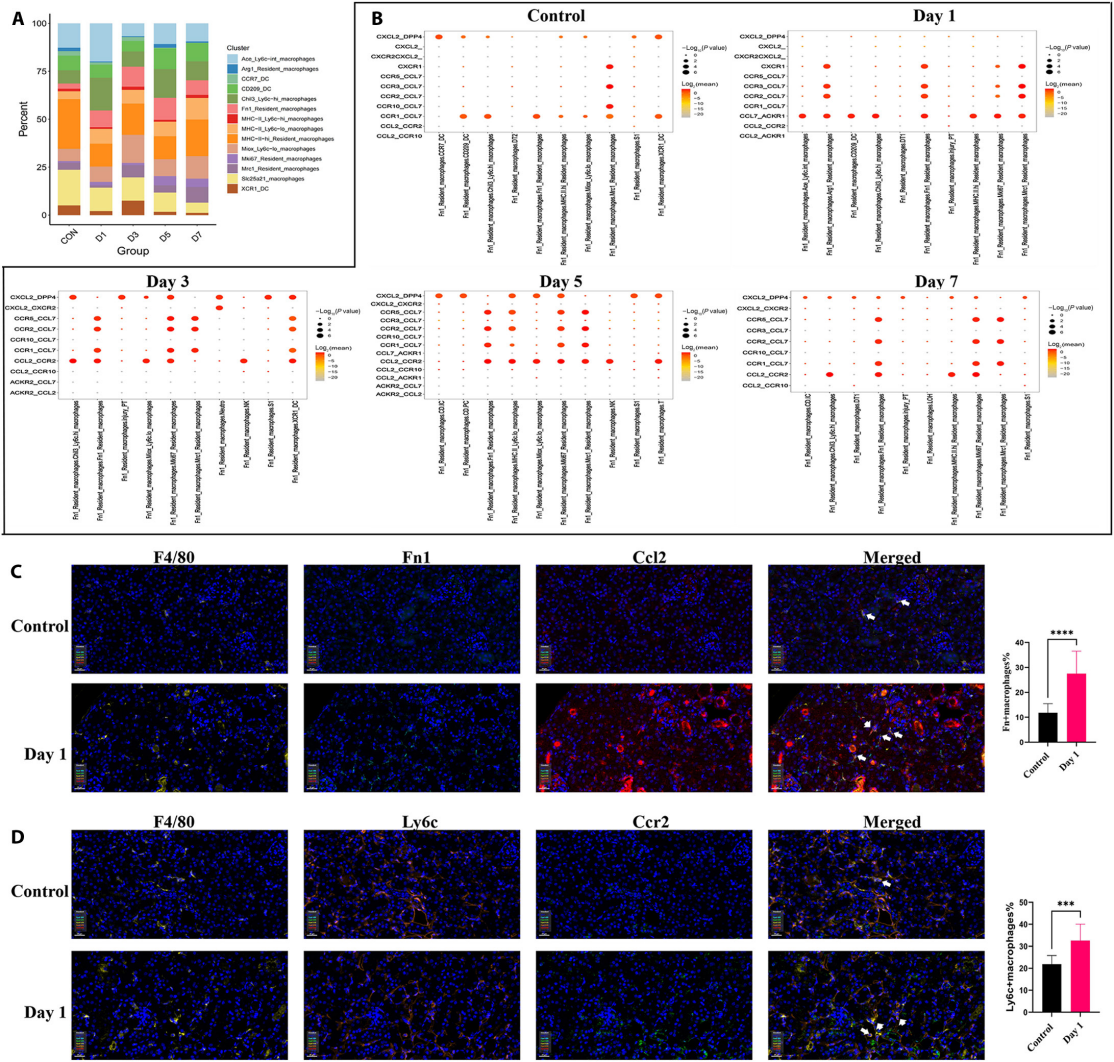
Proportional abundance of each monocyte suclusters and ligand–receptor pairs from chemokines in Fn1 RMs. (A) Connected bar plots showing the proportional abundance of each monocyte subclusters at different days. (B) Dot plots displaying the significant representative ligand–receptor pairs from chemokines in Fn1 RMs to receptors in other cell subtypes at different days. (C) Representative multiple fluorescent of F4/80, Fn1, and CCL2 in kidney sections. F4/80 (yellow), Fn1 (cyan), and CCL2 (red). Scale bar, 20 μm. (D) Representative multiple fluorescent of F4/80, Ly6C, and CCR2 in kidney sections. F4/80 (yellow), Ly6C (orange), and CCR2 (green). Scale bar, 20 μm.

On day 1 post-oxaluria, the number of Chil3 Ly6c^hi^ and Ace Ly6c^int^ macrophages [34,39] increased in the kidneys (Fig. 4A); these cells were considered monocyte-derived infiltrating macrophages. The typical monocyte marker *Ly6c2* [34], whose representative genes are *Chil3* and MHC-II genes, was highly expressed in 2 clusters (Fig. 3A and B). *Ly6c2* expression decreased in Ace Ly6c^int^ macrophages (Fig. 3A and B), and the key markers for this cluster included *Ace* and *Cd74*. The lowest *Ly6c2* expression (found in Ly6c^lo^ macrophages [34,40]) (Fig. 3A and B) occurred in 2 clusters that expressed the marker genes *Miox* and MHC-II genes. RNA velocity analysis revealed a developmental trend from Miox Ly6c^lo^ into Chil3 Ly6c^hi^ macrophages (Fig. 3D and Table S5). Miox Ly6c^lo^ and Chil3 Ly6c^hi^ macrophages played additional roles in cytoplasmic translation and ribosomal small subunit biogenesis in the control group (Fig. S13A and B). However, Chil3 Ly6c^hi^ macrophages were enriched in granulocyte chemotaxis and migration on day 5 (Fig. S13C).

**Fig. 5.**
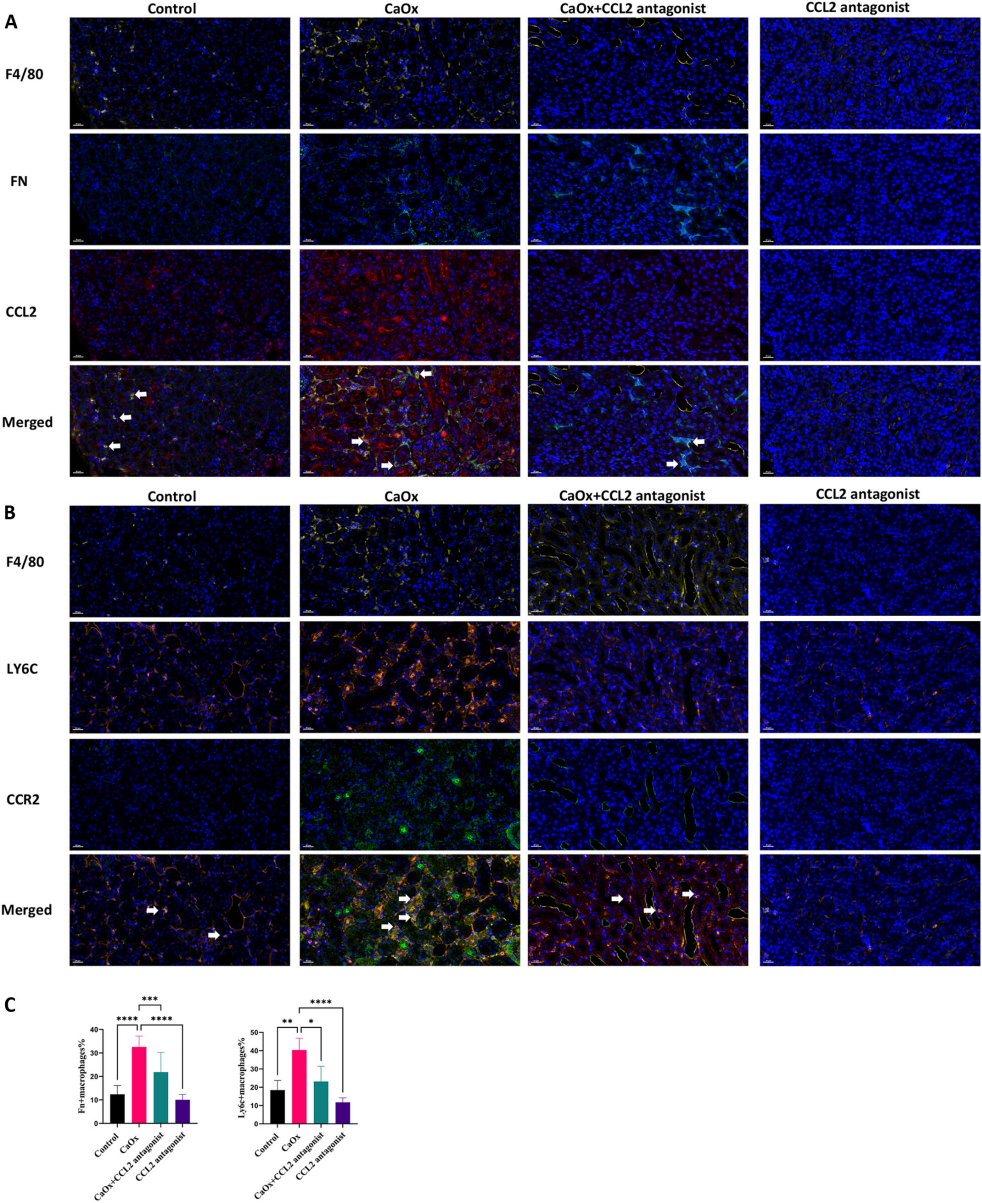
Representative immunofluorescence images of kidney section after mice were treated CCL2 antagonist. (A) Representative multiple fluorescent of F4/80 (yellow), Fn1 (cyan), and CCL2 (red) in kidney sections among control group, CaOx group, CCL2 antagonist in combination with CaOx group, and CCL2 antagonist group. Scale bar, 20 μm. (B) Representative multiple fluorescent of F4/80 (yellow), Ly6C (orange), CCR2 (green), and DAPI (blue) for nuclei in kidney sections among control group, CaOx group, CCL2 antagonist in combination with CaOx group, and CCL2 antagonist group. Scale bar, 20 μm.(C) Fluorescence statistical bar chart. Left panel: statistical chart of Fn^+^ macrophages in (A); right panel: statistical chart of Ly6c^+^ macrophages in (B).

Interestingly, after glyoxylate administration, the expression of many chemokines, such as *Ccl9*, *Ccl2*, *Ccl6*, and *Ccl7*, increased in Fn1 RMs (Fig. 3E), enhancing the interaction between monocyte-derived infiltrated macrophages and RMs through the CCL2–CCR2 and CCL7–CCR2 axes (Fig. 4B). In accordance with previous findings, the number of Fn1 macrophages expressing CCL2 and that of Ly6c macrophages expressing CCR2 increased in the kidneys on day 1 post-hyperoxaluria (Fig. 4C and D). Then, we blocked the CCL2 pathway by using emapticap pegol sodium, an inhibitor of CCL2 [41]. After blocking the CCL2, Ly6c^hi^ infiltrating macrophages were reduced in the mouse kidneys, and the CCR2 expression also decreased in Ly6c^hi^ infiltrating macrophages (Fig. 5A and B). These results indicate that Fn1 RMs expressed chemokines to recruit infiltrating macrophages to the injured kidney area via the CCL2, thereby facilitating an inflammatory response in the kidneys.

### Macrophages mediated CCL2 expression through the CD44–Akt pathway

**Fig. 6.**
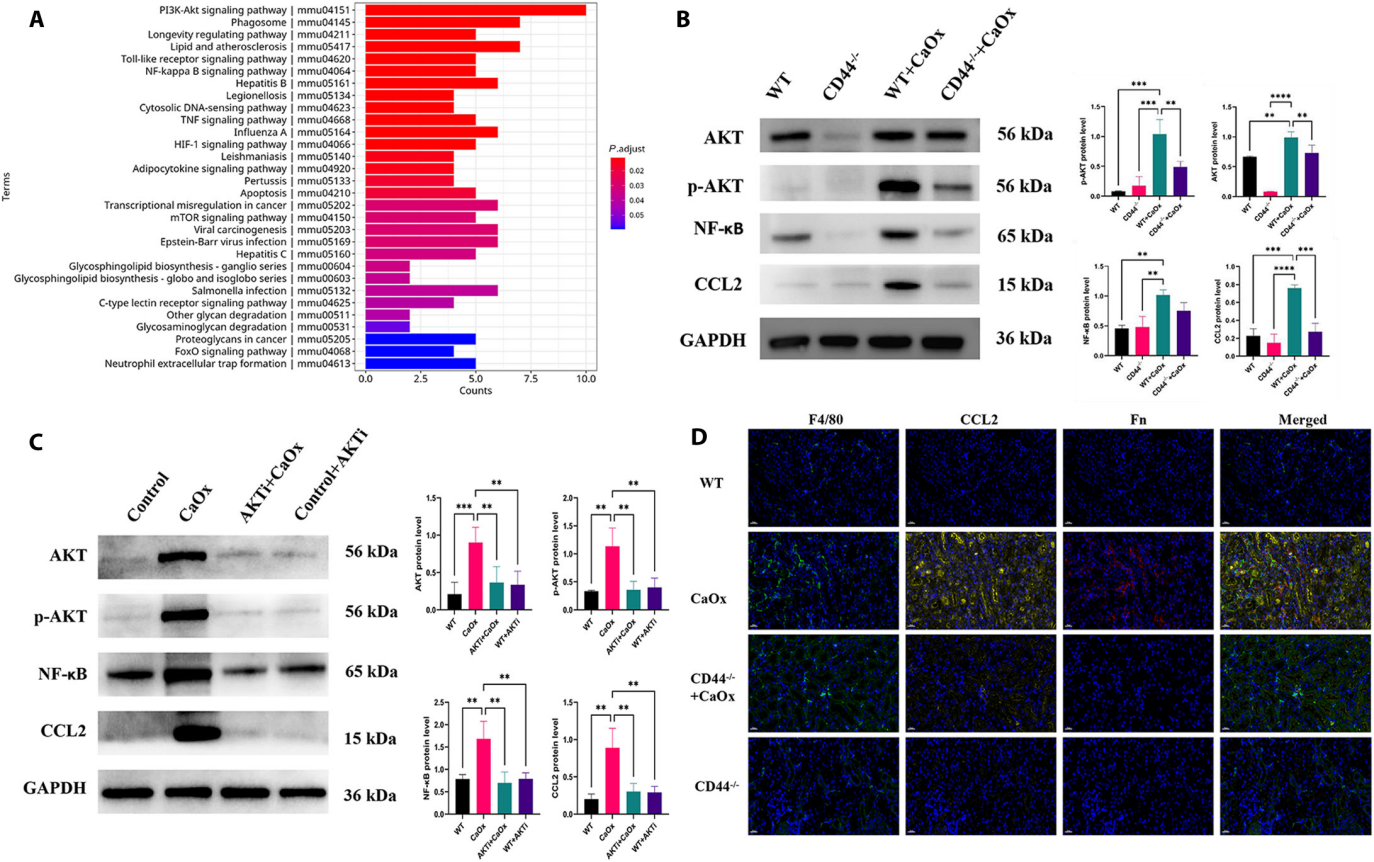
Macrophages mediated CCL2 secretion through the CD44–AKT pathway. (A) KEGG terms enriched in Fn1 RMs. (B) Western blot analysis of the protein levels of AKT, p-AKT, NF-κB, and CCL2 in CD44^−/−^ BMDMs treated with COM (calcium oxalate monohydrate) for 48 h. (C) Western blot analysis of the protein levels of AKT, p-AKT, NF-κB, and CCL2 in BMDMs treated with COM or combined AKT antagonist for 48 h. Untreated wild-type (WT) BMDMs served as controls. Relative quantification of the data (left panel) presented in bar charts (right panel). The data are presented as mean ± SD. Significance was determined by one-way analysis of variance (ANOVA) with Tukey’s multiple comparisons test, *n* = 3 biologically independent samples, ***P < 0.001, **P < 0.01. (D) Representative multiple fluorescent of F4/80 (green), CCL2 (yellow), and Fn (red) in kidney. DAPI (blue) for nuclei. Scale bar, 20 μm.

In previous described results, we found that the renal tubular epithelial cells mainly communicated with monocytes through SPP1–CD44 (Fig. 2E and Fig. S10). We also found that the Kyoto Encyclopedia of Genes and Genomes (KEGG) terms related to the phosphatidylinositol 3-kinase (PI3K)–Akt signaling pathway enriched in Fn1 RMs (Fig. 6A). Then, we supposed that macrophages were activated though CD44, and mediated CCL2 secretion through the PI3K/Akt pathway. We isolated and cultured bone marrow-derived macrophages (BMDMs) from CD44 knockout mice and then stimulated with CaOx crystals. The results showed that macrophages increased Fn1 expression after CaOx crystal stimulation (Fig. S14). Macrophages exhibited up-regulation of AKT, p-AKT, and nuclear factor κB (NF-κB) expression when stimulated with CaOx crystals (Fig. 6B). In contrast, CD44^−/−^ macrophages demonstrated down-regulated expression of AKT, p-AKT, NF-κB, and CCL2 following stimulation with CaOx crystals (Fig. 6B). Then, we treated macrophages with CaOx crystals and AKT antagonist. Similarly, following CaOx crystal stimulation, macrophages exhibited increased expression of AKT, p-AKT, NF-κB, and CCL2. However, when treated with an AKT antagonist, the expression of AKT, p-AKT, NF-κB, and CCL2 in macrophages was reduced upon CaOx crystal stimulation (Fig. 6C). Furthermore, CCL2 expression of Fn1^+^ macrophages decreased in kidneys of CD44^−/−^ mice administered glyoxylate when compared with wild-type mice (Fig. 6D). These results indicated that macrophages mediated CCL2 secretion through the CD44–PI3K/Akt pathway.

### Blocking CCL2 could decrease renal tubule injury and reduced CaOx deposition in mouse kidneys

In the previous study, we found that some parts of PTs were injured in the progression of CaOx formation; however, we also found that other renal tubules exhibited injury, such as the LOH and DTs. After glyoxylate administration, the LOH highly expressed *Lcn2* and *Clu* (Fig. S15A), and the expression of *Lcn2* and *Clu* increased in DTs (expressing Slc12a3; Fig. S15B). Then, we detected the expression of LCN2 and that of CLU in the LOH and DTs of glyoxylate-treated kidneys, as determined by the multiple fluorescent immunohistochemical (IHC) results. The LCN2 and CLU expression increased in the loops of Henle and DTs as the formation of CaOx crystals progressed (Fig. S16A and B). Furthermore, the LCN2, CLU, and CCL2 levels were up-regulated in the urine of patients with CaOx stones (Fig. S15C).

**Fig. 7.**
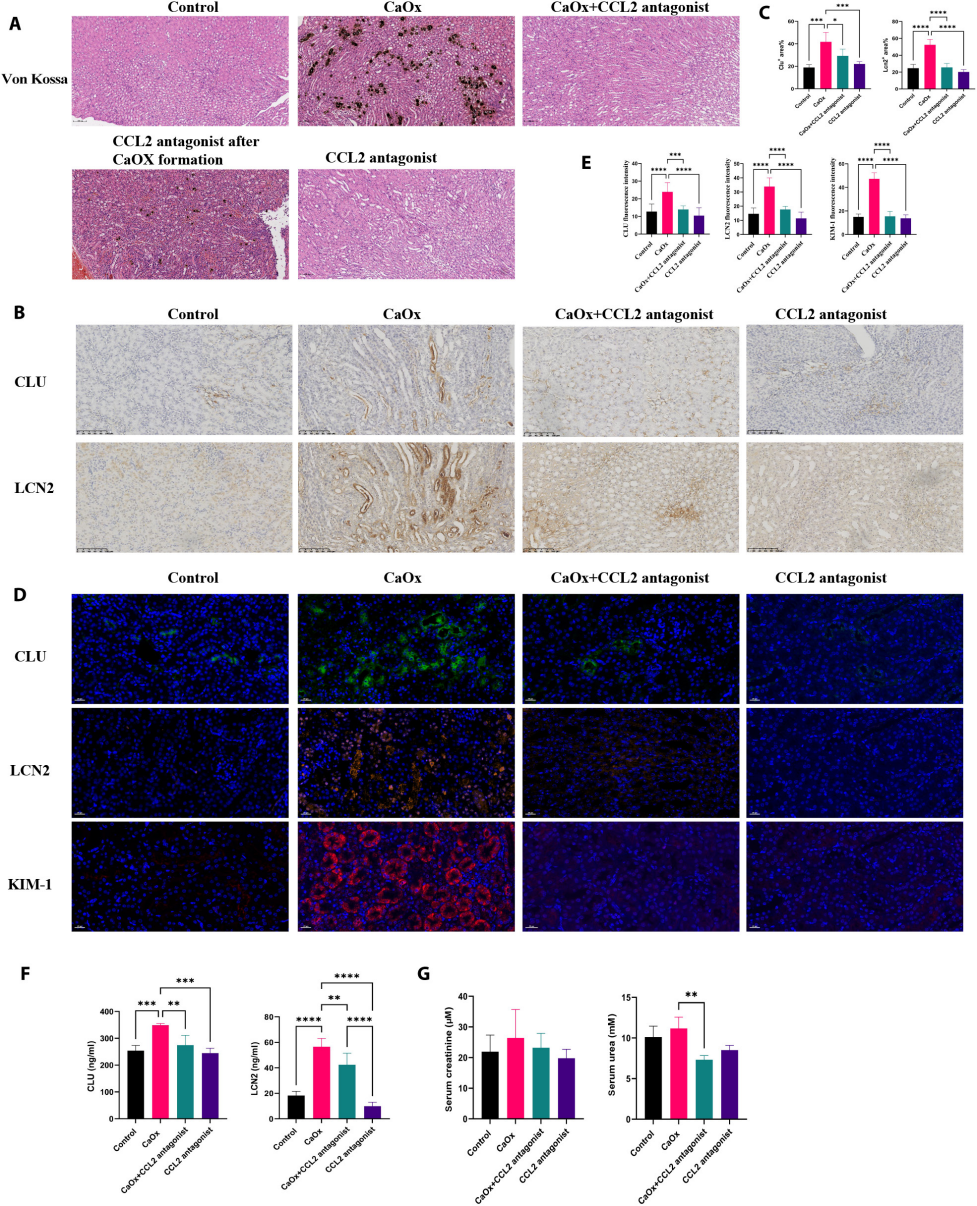
Injury marker expression in kidney. (A) Representative images of H&E and VK staining of kidney section after mice were treated CCL2 antagonist. CCL2 antagonist after CaOx formation group: Mice were administrated with injection of glyoxylate for 3 d and then received a CCL2 antagonist for 4 d. CaOx depositions were black or brown dot plots. Scale bar of H&E and VK staining, 100 μm. (B) IHC staining of CLU and LCN2 in mouse kidneys. (C) Statistical results of (B). (D) Representative CLU (green), LCN2 (orange), and KIM-1 (red) stainings of kidney sections in control group, CaOx group, CCL2 antagonist in combination with CaOx group, and CCL2 antagonist group. Scale bar of immunofluorescence, 20 μm. (E) Statistical results of (D). (F) Level of urine CLU and LCN2 among different groups. (G) Serum creatinine and urea in mice were detected. Data are expressed as mean ± SD. *P < 0.05, **P < 0.01, and ***P < 0.001.

CaOx crystal deposition decreased in the mouse kidneys when CCL2 was inhibited (Fig. 7A). However, CCL2 blockage after crystal formation could not prevent crystal deposition in kidney but could decrease the crystal deposition in the kidney (Fig. 7A). Furthermore, CCL2 blockage reduced injury marker expression in kidney, such as CLU, LCN2, and KIM-1 (Fig. 7A and B), as well as the level of CLU and LCN2 secretion in mouse urine and serum urea (Fig. 7C and D). The findings suggest that suppression of CCL2 effectively reduced the infiltration of immune cells, mitigated renal tubular injury stemming from inflammation, and lessened the accumulation of renal CaOx crystals.

## Discussion

Comprehensively analyzing the accumulation of renal crystals to stones in humans is challenging. Although studies on human kidney specimens have been conducted [32,42,43], the limitations imposed by the site of renal tissue acquisition and the timing of tissue collection during disease progression still prevent us from fully understanding the pathogenic mechanisms underlying this condition. The application of murine models allows the identification of renal characteristics at different stages of renal CaOx crystal formation; however, this strategy is still limited since it does not reflect the full spectrum of renal phenotypes found in patients with oxalate-related crystal nephropathy. scRNA-seq provides a robust and unbiased approach for surveying kidney responses during CaOx crystal formation. Here, we employed scRNA-seq to thoroughly analyze the gene expression patterns of various renal cell types in mice at different time points following glyoxylate administration, offering a valuable resource for future research.

We identified one PT subcluster that highly expressed HAVCR1, known as KIM-1, which was defined as the injured PT and showed an increased expression during CaOx crystal formation in mouse kidneys expressing *Spp1*, *Fgg*, *Fga*, and *Fgb*. El-Achkar and colleagues [43] reported that multiple cell types in human renal papillae with stones expressed increased levels of *Spp1*. Additionally, in a rat model, *Spp1* expression was significantly increased in PTs and immune cells [44]. Interestingly, *Spp1*, *Fgg*, *Fga*, and *Fgb* were also found in the stone matrix. These genes were identified by Witzmann et al. [45], who presented the 50 most abundant proteins in kidney stones, similar to what Canales et al. [46] reported. Notably, our findings extend earlier work by demonstrating that injured PTs not only produce stone matrix proteins but also activate proinflammatory and profibrotic genes (*Cxcl1*, *Tnfrsf12a*, *Nkfb2*, and *Pdgfb*), creating a microenvironment that amplifies immune cell recruitment and crystal aggregation. This dual role of injured PTs, as both contributors to stone matrix formation and initiators of inflammation, mirrors observations in chronic kidney disease models, where stressed tubular cells adopt a secretory phenotype that drives fibrosis [47–49]. As CaOx crystals form in the kidneys, the interaction signals between injured PTs and immune cells increase, further contributing to stone formation.

Macrophages, which are immune cells, are involved in various aspects of the kidney stone formation and resolution processes [7,50]. Crystal nephropathy can trigger an inflammatory response [51–53], and macrophages are key immune cells involved in this process [53,54] since they are attracted to the sites of injury or inflammation in the kidneys and release inflammatory mediators to recruit other immune cells. Our single-cell data reveal that injured PTs engage in crosstalk with macrophages via SPP1–CD44, consistent with the findings of Yu and colleagues [53] in crystal nephropathy models. Crystal retention in kidney may depend on the expression of CD44 in injury cells [55,56]. In our study, the expression of CD44 was mostly on macrophages. In one study of non-alcoholic steatohepatitis, CD44 has been reported to be associated with macrophage infiltration and CCL2 expression [57]. Go Kuwahara et al. [58] reported that adventitial cells in CD44 knockout mice exhibited reduced expression of CCL2 protein and mRNA compared to those in wild-type mice. However, we extend these observations by demonstrating that Fn1^+^ RMs serve as the primary source of CCL2 during early crystal deposition, recruiting infiltrating macrophages through CD44–AKT-dependent signaling. CaOx crystals activate AKT pathways in wild-type macrophages, driving CCL2 expression, whereas CD44^−/−^ macrophages exhibit blunted AKT activation and reduced chemokine output. These results provide a novel link between CD44, a hyaluronan receptor implicated in cell matrix interactions, and chemokine-driven immune recruitment in crystal nephropathy.

CCL2 levels are markedly elevated in both the papillary tips and urine of patients with nephrolithiasis [59]. Our study also reported that urine CCL2 levels were up-regulated in patients with CaOx stones. This finding suggests a potential role for CCL2 in the pathogenesis of CaOx nephrolithiasis. The up-regulation of CCL2 in urine may indicate an inflammatory response associated with stone formation. Furthermore, the presence of elevated CCL2 levels in papillary tips implies a local inflammatory milieu within the kidney, which could contribute to the development and progression of nephrolithiasis. In the study of Wang et al. [60], CCR2, a receptor for CCL2, was evaluated on kidney stone. They discovered that the CCR2 antagonist suppressed CCL2 expression, prevented THP-1 polarization toward the M1 phenotype, and mitigated CaOx-induced damage to HK-2 cells [60]. Dong et al. [61] demonstrated that the CCL2–CCR2 axis on macrophages influences the progression of chronic obstructive pulmonary disease via the PI3K–AKT signaling pathway. In this study, CCL2 inhibition significantly attenuated tubular injury markers, reduced macrophage infiltration, and diminished CaOx crystal deposition. This aligns with clinical evidence of elevated urinary CCL2 in nephrolithiasis patients [59], suggesting that targeting CCL2 could disrupt the feedforward loop of crystal-induced inflammation. Notably, the reduction in Clusterin, a stress protein with paradoxical roles in cytoprotection and matrix stabilization [46,62–64], highlights the dual benefit of CCL2 blockade in mitigating both injury and stone progression. Future studies are warranted to investigate the specific mechanisms by which CCL2 influences stone formation and to explore its potential as a therapeutic target for the management of nephrolithiasis.

**Fig. 8.**
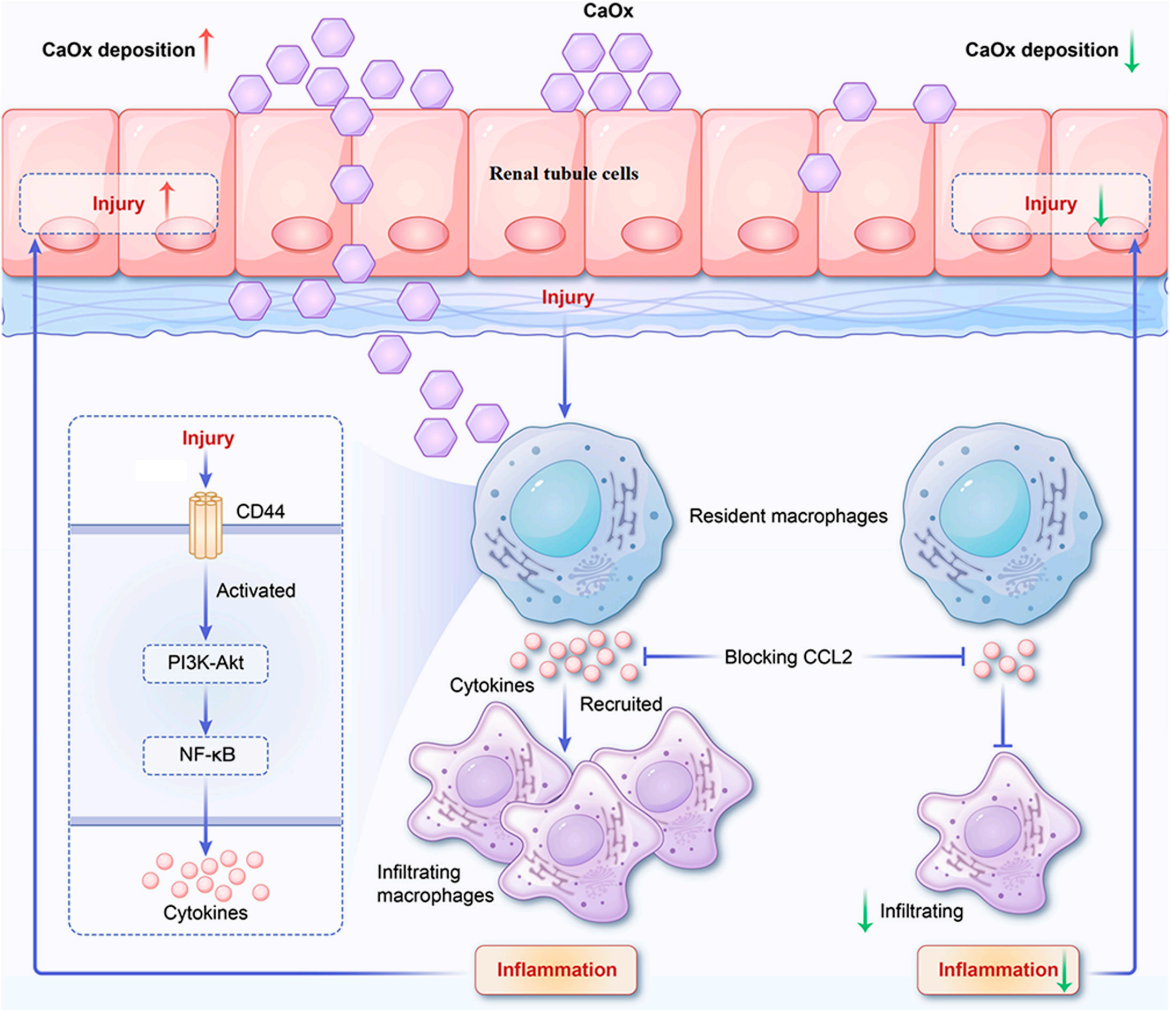
Schematic overview of the current study.

In summary, we presented a detailed atlas of renal cells to clarify the phenotypic characteristics, functions, and interactions between renal tubules and immune cells during CaOx crystal formation in mouse kidneys. scRNA-seq analysis was used to identify the characteristics of injured PTs and revealed that LCN2 and CLU, previously identified in the urine of patients with CaOx stones, may serve as injury markers of the LOH, DTs, and collecting ducts during the progression of CaOx stone disease. Injured PTs express proinflammatory and pro-fibrotic genes that interact with monocytes. Activated RMs recruit infiltrating macrophages to the injured area via the CD44–Akt–CCL2 pathway, thereby facilitating an inflammatory response within the kidneys (Fig. 8). This study sought to enhance our understanding of normal mouse kidney function and the development of CaOx stone disease while also identifying potential cell types and target genes that could be crucial for treating CaOx stone disease in the kidneys.

## Materials and Methods

### Animal study

C57BL/6J mice (male, 8 weeks) were acquired from Chengdu Dossy Experimental Animals Co. Ltd. CD44^−/−^ mice based on CRISPR/Cas9 technology were acquired from GemPharmatech Co. Ltd. This research received approval from the Animal Research Ethics Committee at the West China Hospital, Sichuan University. The mice were maintained in a specific pathogen-free (SPF) environment, with the temperature kept between 20 and 26 °C and relative humidity at 60%. They had unrestricted access to sterilized food and water. The lighting schedule consisted of 12 h of light followed by 12 h of darkness [65]. Mice were administered a single intraperitoneal injection of glyoxylate at a dose of 80 mg/kg (260150, Sigma-Aldrich, Shanghai, China) for 7 consecutive days for the CaOx crystal nephropathy model [66,67]. On days 0, 1, 3, 5, and 7, the mouse kidneys were obtained for scRNA-seq (*n* = 6).

The CCL2 antagonist group received a CCL2 antagonist [68–70] (HY-B0498, Bindarit, 50 mg/kg, MCE, Shanghai, China) combined with the intraperitoneal injection of glyoxylate. On day 8, the kidneys were collected from the mice in each group (*n* = 6).

### Single-cell RNA sequencing

The kidneys digested into cell suspensions were described in the previous study, which were placed in Dulbecco’s modified Eagle’s medium (DMEM) and cut into pieces using collagenase IV (1 mg/ml; Merck, Shanghai, China) and deoxyribonuclease (DNase) I (100 μg/ml, Merck) at 37 °C for 30 min [71,72]. Digested renal cell suspensions with a concentration of 1,000 cells/μl, with cell viability greater than 80%, were prepared and sequenced using 10x Genomics Chromium (Single Cell 3′ library and Gel Bead Kit v3) in accordance with the manufacturer’s instructions as described in a previous study [73]. The samples were sequenced on an Illumina Novaseq6000 instrument using 150 base paired-end reads.

### Sequencing data processing and analysis

To harmonize cells across different samples, we can employ computational methods designed to correct batch effects in scRNA-seq data. In the subsequent sections, we will utilize functions from the Harmony package [74] to address these batch effects.

Cell Ranger was used to process the raw data [which were uploaded to National Center for Biotechnology Information (NCBI): PRJNA1062071]. The Cell Ranger “count” module was used for alignment, filtering, barcode counting, and UMI (unique molecular identifier) counting to generate a feature-barcode matrix and identify the clusters. Cells were deemed low quality and excluded from the analysis if they had fewer than 1,000 UMI counts, less than 200 or more than 10,000 gene counts, or if over 25% of their UMI counts originated from mitochondrial genes. For dimensionality reduction, principal components analysis (PCA) was utilized, and the resulting data were visualized using “UMAP” R packages.

GO analysis, as well as KEGG and Reactome enrichment analyses of the cluster markers, was executed using KOBAS software. GSEA was conducted using version 2.2.2.4 of the GSEA software.

Single-cell trajectories were constructed using the “Monocle” R package, which introduces the pseudo-time. The gene filtering process adhered to the following criteria: They were required to be expressed in over 10 cells, have an average expression level greater than 0.1, and exhibit a *q* value below 0.01.

The analysis of cell–cell interaction was based on the receptor and ligand expressions. CellPhoneDB 2 is a computational analysis tool based on Python, designed for the molecular-level analysis of cell–cell communication [75,76].

### Histological analysis and immunofluorescence staining

The kidneys were fixed, embedded in paraffin, and sectioned into 4-μm-thick slices [71]. H&E staining, along with VK staining, was performed according to the manufacturer’s instructions for the respective staining kits (Solarbio, China) [71].

For the multiple immunofluorescence assays, the kidney sections were stained using Opal 6-Plex detection kits (Akoya Biosciences, MA, USA) according to the manufacturer’s protocol. Kidney sections were incubated with anti-lipocalin 2 (1:100; Bioss, Beijing, China), anti-CLU (1:100; Affbiotech, Jiangsu, China), anti-SLC12A3 (1:200; Affbiotech), anti-NKCC2 (SLC12A1) (1:2,000; Proteintech, Wuhan, China), anti-UMOD (1:1,000; Proteintech), anti-AQP2 (1:1,000; Proteintech), anti-ATP6V1B1 (1:400; Proteintech), anti-fibronectin (1:200, Abcam, Shanghai, China), anti-F4/80 (1:200, Abcam), anti-CD68 (1:200, Abcam), anti-MCP1 (1:400, Affbiotech), anti-CCR2 (1:200, Affbiotech), and anti-Ly6C (1:400, Abcam). For the 2-color immunofluorescence assay, the kidney sections were subjected to incubation with the primary antibodies, anti-TIM-1 (1:100; GeneTex, CA, USA) and anti-LTL (*Lotus tetragonolobus* lectin; 1:50, Vectorlabs, Newark, CA), at 4 °C for 24 h. The slides were incubated with fluorescent secondary antibody [streptavidin fluorescein isothiocyanate (FITC), Alexa Fluor 594, 1:1,000, Thermo Fisher Scientific, Shanghai, China] for 4 h at room temperature.

### BMDM isolation and culture

BMDMs were isolated from the tibias and femurs of C57BL/6J mice and CD44^−/−^ mice. The cells were cultured in DMEM (Gibco, USA) supplemented with 10% fetal bovine serum (Gibco), 50 U/ml penicillin, 50 μg/ml streptomycin, and 20 ng/ml murine macrophage colony-stimulating factor (Novoprotein, Shanghai, China) [77,78] for 6 d. Mature BMDMs were used for further experiments. BMDMs were stimulated with CaOx crystals at a ratio of crystals per macrophage, with or without AKT antagonist (Akt1/Akt2-IN-1, HY-50862, 5 μm, MCE, Shanghai, China).

### Western blotting

The samples of macrophages were lysed in radioimmunoprecipitation assay (RIPA) buffer (Beyotime, Shanghai, China), which included phosphatase and protease inhibitors, for a period of 30 min. The total protein concentration was measured using a BCA (bicinchoninic acid) kit (P0010, Beyotime). Equal quantities of protein were then separated by 8% to 12% sodium dodecyl sulfate–polyacrylamide gel electrophoresis (SDS-PAGE) and subsequently transferred to PVDF (polyvinylidene difluoride, Millipore, USA) membranes. The PVDF membranes were pretreated with QuickBlock Blocking Buffer (P0235, Beyotime) prior to incubation with the respective primary antibodies: anti-AKT (ET1609-51, HUABIO, Hangzhou, China), anti-phospho-AKT (Ser^473^) (ET-1607-73, HUABIO), anti-NF-κB (ET1603-12, HUABIO), and CCL2 (83157-5-RR, Proteintech) antibodies. The enzyme-tagged secondary antibodies were applied to the PVDF membrane for a duration of 1 h at room temperature. Subsequently, the protein bands were illuminated using an advanced chemiluminescence detection kit and visualized with an imaging apparatus (Bio-Rad, ChemiDoc MP).

### ELISA assay

The study involving patient samples was approved by the Biomedical Research Ethics Committee (West China Hospital, Sichuan University). Midstream specimens of urine in the morning were collected from patients with CaOx stones and healthy controls to detect CLU and LCN2 by using the human Clusterin ELISA (enzyme-linked immunosorbent assay) Kit and human Lipocalin-2 ELISA Kit (Boster Biological Technology, Wuhan, China) according to the manufacturer’s instructions. Mice serum and urine were collected from CaOx mice and control mice to detect serum creatinine (biochemical analyzer), CLU, and LCN2 by ELISA Kit (Boster Biological Technology).

### Statistical analysis

All statistical analyses were performed using GraphPad Prism, version 9.5.1. The data are expressed as the mean ± standard deviation (SD). Comparisons between the 2 groups were conducted using Student’s *t* test. Statistically significant differences are denoted as *P < 0.05 or **P < 0.01.

## Data Availability

The scRNA-seq data were deposited in the NCBI’s Sequence Read Archive (SRA, BioProject PRJNA1062071).

## References

[1] Mulay SR, Anders H-J. Crystal nephropathies: Mechanisms of crystal-induced kidney injury. Nat Rev Nephrol. 2017;13(4):226–240. 28218266 10.1038/nrneph.2017.10

[2] Pathan SA, Mitra B, Cameron PA. A systematic review and meta-analysis comparing the efficacy of nonsteroidal anti-inflammatory drugs, opioids, and paracetamol in the treatment of acute renal colic. Eur Urol. 2018;73(4):583–595. 29174580 10.1016/j.eururo.2017.11.001

[3] Dursun M, Ozbek E, Otunctemur A, Sahin S, Cakir SS. Clinical presentation of urolithiasis in older and younger population. Arch Ital Urol Androl. 2014;86(4):249–252. 25641444 10.4081/aiua.2014.4.249

[4] Gu Y, Shen Y, Chen W, He H, Ma Y, Mei X, Ju D, Liu H. Protective effects of interleukin-22 on oxalate-induced crystalline renal injury via alleviating mitochondrial damage and inflammatory response. Appl Microbiol Biotechnol. 2022;106(7):2637–2649. 35294590 10.1007/s00253-022-11876-4

[5] Demoulin N, Aydin S, Gillion V, Morelle J, Jadoul M. Pathophysiology and management of hyperoxaluria and oxalate nephropathy: A review. Am J Kidney Dis. 2022;79(5):717–727. 34508834 10.1053/j.ajkd.2021.07.018

[6] Desenclos J, Forté V, Clément C, Daudon M, Letavernier E. Pathophysiology and management of enteric hyperoxaluria. Clin Res Hepatol Gastroenterol. 2024;48(5): Article 102322. 38503362 10.1016/j.clinre.2024.102322

[7] Khan SR, Canales BK, Dominguez-Gutierrez PR. Randall’s plaque and calcium oxalate stone formation: Role for immunity and inflammation. Nat Rev Nephrol. 2021;17(6):417–433. 33514941 10.1038/s41581-020-00392-1

[8] Coe FL, Worcester EM, Evan AP. Idiopathic hypercalciuria and formation of calcium renal stones. Nat Rev Nephrol. 2016;12(11):519–533. 27452364 10.1038/nrneph.2016.101 PMC5837277

[9] Sorokin I, Mamoulakis C, Miyazawa K, Rodgers A, Talati J, Lotan Y. Epidemiology of stone disease across the world. World J Urol. 2017;35(9):1301–1320. 28213860 10.1007/s00345-017-2008-6

[10] Scales CD, Smith AC, Hanley JM, Saigal CS, Urologic Diseases in America Project. Prevalence of kidney stones in the United States. Eur Urol. 2012;62(1):160–165. 22498635 10.1016/j.eururo.2012.03.052 PMC3362665

[11] Liu Y, Chen Y, Liao B, Luo D, Wang K, Li H, Zeng G. Epidemiology of urolithiasis in Asia. Asian J Urol. 2018;5(4):205–214. 30364478 10.1016/j.ajur.2018.08.007 PMC6197415

[12] Monga M, Murphy M, Paranjpe R, Cutone B, Eisner B. Prevalence of stone disease and procedure trends in the United States. Urology. 2023;176:63–68. 37062518 10.1016/j.urology.2023.03.040

[13] Stamatelou K, Goldfarb DS. Epidemiology of kidney stones. Healthcare. 2023;11(3):424. 36766999 10.3390/healthcare11030424 PMC9914194

[14] Filler G, Dave S, Ritter V, Ross S, Viprakasit D, Hatch JE, Bjazevic J, Burton J, Gilleskie D, Gilliland J, et al. In focus: Perplexing increase of urinary stone disease in children, adolescent and young adult women and its economic impact. Front Med. 2023;10:1272900. 10.3389/fmed.2023.1272900 PMC10626457 37937142

[15] Singh P, Harris PC, Sas DJ, Lieske JC. The genetics of kidney stone disease and nephrocalcinosis. Nat Rev Nephrol. 2022;18(4):224–440. 34907378 10.1038/s41581-021-00513-4

[16] Wróbel G, Kuder T. The role of selected environmental factors and the type of work performed on the development of urolithiasis—A review paper. Int J Occup Med Environ Health. 2019;32(6):761–775. 31625537 10.13075/ijomeh.1896.01491

[17] Siener R. Nutrition and kidney stone disease. Nutrients. 2021;13(6):1917. 34204863 10.3390/nu13061917 PMC8229448

[18] Shang W, Li Y, Ren Y, Yang Y, Li H, Dong J. Nephrolithiasis and risk of hypertension: A meta-analysis of observational studies. BMC Nephrol. 2017;18(1):344. 29187160 10.1186/s12882-017-0762-8 PMC5708110

[19] Mulay SR, Honarpisheh MM, Foresto-Neto O, Shi C, Desai J, Zhao ZB, Marschner JA, Popper B, Buhl EM, Boor P, et al. Mitochondria permeability transition versus necroptosis in oxalate-induced AKI. J Am Soc Nephrol. 2019;30(10):1857–1869. 31296606 10.1681/ASN.2018121218 PMC6779355

[20] Lu C-L, Teng T-Y, Liao M-T, Ma M-C. TRPV1 hyperfunction contributes to renal inflammation in oxalate nephropathy. Int J Mol Sci. 2021;22(12):6204. 34201387 10.3390/ijms22126204 PMC8228656

[21] Shi Y-S, Yang T-N, Wang Y-X, Ma X-Y, Liu S, Zhao Y, Li J-L. Melatonin mitigates atrazine-induced renal tubular epithelial cell senescence by promoting Parkin-mediated mitophagy. Research. 2024;7:0378. 38766643 10.34133/research.0378 PMC11098712

[22] Khan SR, Pearle MS, Robertson WG, Gambaro G, Canales BK, Doizi S, Traxer O, Tiselius HG. Kidney stones. Nat Rev Dis Primer. 2016;2:16008. 10.1038/nrdp.2016.8 PMC5685519 27188687

[23] Convento MB, Pessoa EA, Cruz E, da Glória MA, Schor N, Borges FT. Calcium oxalate crystals and oxalate induce an epithelial-to-mesenchymal transition in the proximal tubular epithelial cells: Contribution to oxalate kidney injury. Sci Rep. 2017;7:45740. 28387228 10.1038/srep45740 PMC5384284

[24] Duan X, Kong Z, Mai X, Lan Y, Liu Y, Yang Z, Zhao Z, Deng T, Zeng T, Cai C, et al. Autophagy inhibition attenuates hyperoxaluria-induced renal tubular oxidative injury and calcium oxalate crystal depositions in the rat kidney. Redox Biol. 2018;16:414–425. 29653411 10.1016/j.redox.2018.03.019 PMC5953241

[25] Mulay SR, Eberhard JN, Desai J, Marschner JA, Kumar SVR, Weidenbusch M, Grigorescu M, Lech M, Eltrich N, Müller L, et al. Hyperoxaluria requires TNF receptors to initiate crystal adhesion and kidney stone disease. J Am Soc Nephrol. 2017;28(3):761–768. 27612997 10.1681/ASN.2016040486 PMC5328164

[26] Aihara K, Byer KJ, Khan SR. Calcium phosphate-induced renal epithelial injury and stone formation: Involvement of reactive oxygen species. Kidney Int. 2003;64(4):1283–1291. 12969146 10.1046/j.1523-1755.2003.00226.x

[27] Kirita Y, Wu H, Uchimura K, Wilson PC, Humphreys BD. Cell profiling of mouse acute kidney injury reveals conserved cellular responses to injury. Proc Natl Acad Sci USA. 2020;117(27):15874–15883. 32571916 10.1073/pnas.2005477117 PMC7355049

[28] Doke T, Abedini A, Aldridge DL, Yang YW, Park J, Hernandez CM, Balzer MS, Shrestra R, Coppock G, Rico JMI, et al. Single-cell analysis identifies the interaction of altered renal tubules with basophils orchestrating kidney fibrosis. Nat Immunol. 2022;23(6):947–959. 35552540 10.1038/s41590-022-01200-7 PMC11783796

[29] Lu Y-A, Liao C-T, Raybould R, Talabani B, Grigorieva I, Szomolay B, Bowen T, Andrews R, Taylor PR, Fraser D. Single-nucleus RNA sequencing identifies new classes of proximal tubular epithelial cells in kidney fibrosis. J Am Soc Nephrol. 2021;32(10):2501–2516. 34155061 10.1681/ASN.2020081143 PMC8722798

[30] Ransick A, Lindström NO, Liu J, Zhu Q, Guo JJ, Alvarado GF, Kim AD, Black HG, Kim J, McMahon AP. Single-cell profiling reveals sex, lineage, and regional diversity in the mouse kidney. Dev Cell. 2019;51(3):399–413.e7. 31689386 10.1016/j.devcel.2019.10.005 PMC6948019

[31] Park J, Shrestha R, Qiu C, Kondo A, Huang S, Werth M, Li M, Barasch J, Suszták K. Single-cell transcriptomics of the mouse kidney reveals potential cellular targets of kidney disease. Science. 2018;360(6390):758–763. 29622724 10.1126/science.aar2131 PMC6188645

[32] Boonla C, Tosukhowong P, Spittau B, Schlosser A, Pimratana C, Krieglstein K. Inflammatory and fibrotic proteins proteomically identified as key protein constituents in urine and stone matrix of patients with kidney calculi. Clin Chim Acta. 2014;429:81–89. 24333391 10.1016/j.cca.2013.11.036

[33] Wang G, Heijs B, Kostidis S, Mahfouz A, Rietjens RGJ, Bijkerk R, Koudijs A, van der Pluijm LAK, van den Berg CW, Dumas SJ, et al. Analyzing cell-type-specific dynamics of metabolism in kidney repair. Nat Metab. 2022;4(9):1109–1118. 36008550 10.1038/s42255-022-00615-8 PMC9499864

[34] Yao W, Chen Y, Li Z, Ji J, You A, Jin S, Ma Y, Zhao Y, Wang J, Qu L, et al. Single cell RNA sequencing identifies a unique inflammatory macrophage subset as a druggable target for alleviating acute kidney injury. Adv Sci. 2022;9(12): Article e2103675. 10.1002/advs.202103675 PMC9036000 35112806

[35] Zimmerman KA, Bentley MR, Lever JM, Li Z, Crossman DK, Song CJ, Liu S, Crowley MR, George JF, Mrug M, et al. Single-cell RNA sequencing identifies candidate renal resident macrophage gene expression signatures across species. J Am Soc Nephrol. 2019;30(5):767–781. 30948627 10.1681/ASN.2018090931 PMC6493978

[36] Huggins DN, LaRue RS, Wang Y, Knutson TP, Xu Y, Williams JW, Schwertfeger KL. Characterizing macrophage diversity in metastasis-bearing lungs reveals a lipid-associated macrophage subset. Cancer Res. 2021;81(20):5284–5295. 34389631 10.1158/0008-5472.CAN-21-0101 PMC8530952

[37] Li R, Ferdinand JR, Loudon KW, Bowyer GS, Laidlaw S, Muyas F, Mamanova L, Neves JB, Bolt L, Fasouli ES, et al. Mapping single-cell transcriptomes in the intra-tumoral and associated territories of kidney cancer. Cancer Cell. 2022;40(12):1583–1599.e10. 36423636 10.1016/j.ccell.2022.11.001 PMC9767677

[38] Conway BR, O’Sullivan ED, Cairns C, O’Sullivan J, Simpson DJ, Salzano A, Connor K, Ding P, Humphries D, Stewart K, et al. Kidney single-cell atlas reveals myeloid heterogeneity in progression and regression of kidney disease. J Am Soc Nephrol. 2020;31(12):2833–2854. 32978267 10.1681/ASN.2020060806 PMC7790206

[39] Abuduyimiti T, Goto H, Kimura K, Oshima Y, Tanida R, Kamoshita K, Leerach N, Abuduwaili H, Oo HK, Li Q, et al. Diabetes accelerates steatohepatitis in mice: Liver pathology and single-cell gene expression signatures. Am J Pathol. 2024;194(5):693–707. 38309428 10.1016/j.ajpath.2024.01.007

[40] Guo W, Li Z, Anagnostopoulos G, Kong WT, Zhang S, Chakarov S, Shin A, Qian J, Zhu Y, Bai W, et al. Notch signaling regulates macrophage-mediated inflammation in metabolic dysfunction-associated steatotic liver disease. Immunity. 2024;57(10):2310–2327.e6. 39317200 10.1016/j.immuni.2024.08.016

[41] Boels MGS, Koudijs A, Avramut MC, Sol WMPJ, Wang G, van Oeveren-Rietdijk A, van Zonneveld A, de Boer HC, van der Vlag J, van Kooten C, et al. Systemic monocyte chemotactic protein-1 inhibition modifies renal macrophages and restores glomerular endothelial glycocalyx and barrier function in diabetic nephropathy. Am J Pathol. 2017;187(11):2430–2440. 28837800 10.1016/j.ajpath.2017.07.020

[42] Daudon M, Frochot V, Bazin D, Jungers P. Drug-induced kidney stones and crystalline nephropathy: Pathophysiology, prevention and treatment. Drugs. 2018;78(2):163–201. 29264783 10.1007/s40265-017-0853-7

[43] Canela VH, Bowen WS, Ferreira RM, Syed F, Lingeman JE, Sabo AR, Barwinska D, Winfree S, Lake BB, Cheng YH, et al. A spatially anchored transcriptomic atlas of the human kidney papilla identifies significant immune injury in patients with stone disease. Nat Commun. 2023;14(1):4140. 37468493 10.1038/s41467-023-38975-8 PMC10356953

[44] Wang Z, Deng Q, Gu Y, Li M, Chen Y, Wang J, Zhang Y, Zhang J, Hu Q, Zhang S, et al. Integrated single-nucleus sequencing and spatial architecture analysis identified distinct injured-proximal tubular types in calculi rats. Cell Biosci. 2023;13(1):92. 37208718 10.1186/s13578-023-01041-3 PMC10197242

[45] Witzmann FA, Evan AP, Coe FL, Worcester EM, Lingeman JE, Williams JC. Label-free proteomic methodology for the analysis of human kidney stone matrix composition. Proteome Sci. 2016;14:4. 26924944 10.1186/s12953-016-0093-x PMC4769560

[46] Canales BK, Anderson L, Higgins L, Slaton J, Roberts KP, Liu N, Monga M. Second prize: Comprehensive proteomic analysis of human calcium oxalate monohydrate kidney stone matrix. J Endourol. 2008;22(6):1161–1167. 18484873 10.1089/end.2007.0440

[47] Baker ML, Cantley LG. Adding insult to injury: The spectrum of tubulointerstitial responses in acute kidney injury. J Clin Invest. 2025;135(6): Article e188358. 40091836 10.1172/JCI188358 PMC11910233

[48] Lieske JC, Deganello S. Nucleation, adhesion, and internalization of calcium-containing urinary crystals by renal cells. J Am Soc Nephrol. 1999;10(Suppl 14):S422–S429. 10541277

[49] Weinmann-Menke J, Gonzalez-Sanchez HM, Iwata Y, Meineck M, Abassi N, Marini F, Granados-Contreras F, Takakura A, Noda M, Kelley VR. Ptprz signaling, tubule- and macrophage-mediated kidney injury, and subsequent CKD. J Am Soc Nephrol. 2025. 10.1681/ASN.0000000640 PMC12187241 39932811

[50] Kumar P, Laurence E, Crossman DK, Assimos DG, Murphy MP, Mitchell T. Oxalate disrupts monocyte and macrophage cellular function via Interleukin-10 and mitochondrial reactive oxygen species (ROS) signaling. Redox Biol. 2023;67: Article 102919. 37806112 10.1016/j.redox.2023.102919 PMC10565874

[51] Capolongo G, Ferraro PM, Unwin R. Inflammation and kidney stones: Cause and effect? Curr Opin Urol. 2023;33(2):129–135. 36562282 10.1097/MOU.0000000000001066

[52] Frasconi TM, Kurts C, Dhana E, Kaiser R, Reichelt M, Lukacs-Kornek V, Boor P, Hauser AE, Pascual-Reguant A, Bedoui S, et al. Renal IL-23-dependent type 3 innate lymphoid cells link crystal-induced intrarenal inflammasome activation with kidney fibrosis. J Immunol. 2024;213(6):865–875. 39072698 10.4049/jimmunol.2400041 PMC11372247

[53] Huang L, Chen W, Tan Z, Huang Y, Gu X, Liu L, Zhang H, Shi Y, Ding J, Zheng C, et al. Mrc1^+^ macrophage-derived IGF1 mitigates crystal nephropathy by promoting renal tubule cell proliferation via the AKT/Rb signaling pathway. Theranostics. 2024;14(4):1764–1780. 38389846 10.7150/thno.89174 PMC10879870

[54] Okada A, Yasui T, Fujii Y, Niimi K, Hamamoto S, Hirose M, Kojima Y, Itoh Y, Tozawa K, Hayashi Y, et al. Renal macrophage migration and crystal phagocytosis via inflammatory-related gene expression during kidney stone formation and elimination in mice: Detection by association analysis of stone-related gene expression and microstructural observation. J Bone Miner Res. 2010;25(12):2701–2711. 20577968 10.1002/jbmr.158

[55] Asselman M, Verhulst A, De Broe ME, Verkoelen CF. Calcium oxalate crystal adherence to hyaluronan-, osteopontin-, and CD44-expressing injured/regenerating tubular epithelial cells in rat kidneys. J Am Soc Nephrol. 2003;14(12):3155–3166. 14638914 10.1097/01.asn.0000099380.18995.f7

[56] Verhulst A, Asselman M, Persy VP, Schepers MSJ, Helbert MF, Verkoelen CF, de Broe ME. Crystal retention capacity of cells in the human nephron: Involvement of CD44 and its ligands hyaluronic acid and osteopontin in the transition of a crystal binding- into a nonadherent epithelium. J Am Soc Nephrol. 2003;14(1):107–115. 12506143 10.1097/01.asn.0000038686.17715.42

[57] Patouraux S, Rousseau D, Bonnafous S, Lebeaupin C, Luci C, Canivet CM, Schneck AS, Bertola A, Saint-Paul MC, Iannelli A, et al. CD44 is a key player in non-alcoholic steatohepatitis. J Hepatol. 2017;67(2):328–338. 28323124 10.1016/j.jhep.2017.03.003

[58] Kuwahara G, Hashimoto T, Tsuneki M, Yamamoto K, Assi R, Foster TR, Hanisch JJ, Bai H, Hu H, Protack CD, et al. CD44 promotes inflammation and extracellular matrix production during arteriovenous fistula maturation. Arterioscler Thromb Vasc Biol. 2017;37(6):1147–1156. 28450292 10.1161/ATVBAHA.117.309385 PMC5467640

[59] Sun AY, Hinck B, Cohen BR, Keslar K, Fairchild RL, Monga M. Inflammatory cytokines in the papillary tips and urine of nephrolithiasis patients. J Endourol. 2018;32(3):236–244. 29338314 10.1089/end.2017.0699

[60] Wang X, Xie L, Liu C. CCR2 antagonist attenuates calcium oxalate-induced kidney oxidative stress and inflammation by regulating macrophage activation. Exp Anim. 2024;73(2):211–222. 38199255 10.1538/expanim.23-0113 PMC11091353

[61] Dong Y, Dong Y, Zhu C, Yang L, Wang H, Li J, Zheng Z, Zhao H, Xie W, Chen M, et al. Targeting CCL2-CCR2 signaling pathway alleviates macrophage dysfunction in COPD via PI3K-AKT axis. Cell Commun Signal. 2024;22(1):364. 39014433 10.1186/s12964-024-01746-z PMC11253350

[62] Cunin P, Beauvillain C, Miot C, Augusto JF, Preisser L, Blanchard S, Pignon P, Scotet M, Garo E, Fremaux I, et al. Clusterin facilitates apoptotic cell clearance and prevents apoptotic cell-induced autoimmune responses. Cell Death Dis. 2016;7(5): Article e2215. 27148688 10.1038/cddis.2016.113 PMC4917652

[63] Jung G-S, Kim M-K, Jung Y-A, Kim HS, Park IS, Min BH, Lee KU, Kim JG, Park KG, Lee IK. Clusterin attenuates the development of renal fibrosis. J Am Soc Nephrol. 2012;23(1):73–85. 22052058 10.1681/ASN.2011010048 PMC3269926

[64] Yang JYC, Sarwal RD, Ky K, Dong V, Stoller M, Sarwal MM, Chi T. Non-radiological assessment of kidney stones using the kidney injury test (KIT), a spot urine assay. BJU Int. 2020;125(5):732–738. 31869527 10.1111/bju.14978

[65] Jin X, Lin T, Yang G, Cai H, Tang B, Liao X, Li H, Chen X, Gong L, Xu H, et al. Use of Tregs as a cell-based therapy via CD39 for benign prostate hyperplasia with inflammation. J Cell Mol Med. 2020;24(9):5082–5096. 32191396 10.1111/jcmm.15137 PMC7205803

[66] Taguchi K, Chen L, Usawachintachit M, Hamamoto S, Kang M, Sugino T, Unno R, Tzou DT, Sherer BA, Okada A, et al. Fatty acid-binding protein 4 downregulation drives calcification in the development of kidney stone disease. Kidney Int. 2020;97(5):1042–1056. 32247632 10.1016/j.kint.2020.01.042

[67] Okada A, Nomura S, Higashibata Y, Hirose M, Gao B, Yoshimura M, Itoh Y, Yasui T, Tozawa K, Kohri K. Successful formation of calcium oxalate crystal deposition in mouse kidney by intraabdominal glyoxylate injection. Urol Res. 2007;35(2):89–99. 17393196 10.1007/s00240-007-0082-8

[68] Shen Z, Kuang S, Zhang M, Huang X, Chen J, Guan M, Qin W, Xu HHK, Lin Z. Inhibition of CCL2 by bindarit alleviates diabetes-associated periodontitis by suppressing inflammatory monocyte infiltration and altering macrophage properties. Cell Mol Immunol. 2021;18(9):2224–2235. 32678310 10.1038/s41423-020-0500-1 PMC8429574

[69] Ni J, Guo T, Zhou Y, Jiang S, Zhang L, Zhu Z. STING signaling activation modulates macrophage polarization via CCL2 in radiation-induced lung injury. J Transl Med. 2023;21(1):590. 37667317 10.1186/s12967-023-04446-3 PMC10476398

[70] Jiang W, Xu T, Song Z, Wang X, Yuan S, Li Q, Wei Y, Wang C, Yang G, Cao J, et al. CCL2 is a key regulator and therapeutic target for periodontitis. J Clin Periodontol. 2023;50(12):1644–1657. 37697486 10.1111/jcpe.13872

[71] Jin X, Jian Z, Chen X, Ma Y, Ma H, Liu Y, Gong L, Xiang L, Zhu S, Shu X, et al. Short chain fatty acids prevent glyoxylate-induced calcium oxalate stones by GPR43-dependent immunomodulatory mechanism. Front Immunol. 2021;12: Article 729382. 34675921 10.3389/fimmu.2021.729382 PMC8523925

[72] Cao Q, Wang Y, Niu Z, Wang C, Wang R, Zhang Z, Chen T, Wang XM, Li Q, Lee VWS, et al. Potentiating tissue-resident type 2 innate lymphoid cells by IL-33 to prevent renal ischemia-reperfusion injury. J Am Soc Nephrol. 2018;29(3):961–976. 29295873 10.1681/ASN.2017070774 PMC5827602

[73] Peng L, Jin X, Li B-Y, Zeng X, Liao BH, Jin T, Chen JW, Gao XS, Wang W, He Q, et al. Integrating single-cell RNA sequencing with spatial transcriptomics reveals immune landscape for interstitial cystitis. Signal Transduct Target Ther. 2022;7(1):161. 35589692 10.1038/s41392-022-00962-8 PMC9120182

[74] Korsunsky I, Millard N, Fan J, Slowikowski K, Zhang F, Wei K, Baglaenko Y, Brenner M, Loh PR, Raychaudhuri S. Fast, sensitive and accurate integration of single-cell data with harmony. Nat Methods. 2019;16(12):1289–1296. 31740819 10.1038/s41592-019-0619-0 PMC6884693

[75] Vento-Tormo R, Efremova M, Botting RA, Turco MY, Vento-Tormo M, Meyer KB, Park JE, Stephenson E, Polański K, Goncalves A, et al. Single-cell reconstruction of the early maternal-fetal interface in humans. Nature. 2018;563(7731):347–353. 30429548 10.1038/s41586-018-0698-6 PMC7612850

[76] Efremova M, Vento-Tormo M, Teichmann SA, Vento-Tormo R. CellPhoneDB: Inferring cell-cell communication from combined expression of multi-subunit ligand-receptor complexes. Nat Protoc. 2020;15(4):1484–1506. 32103204 10.1038/s41596-020-0292-x

[77] Deng A, Fan R, Hai Y, Zhuang J, Zhang B, Lu X, Wang W, Luo L, Bai G, Liang L, et al. A STING agonist prodrug reprograms tumor-associated macrophage to boost colorectal cancer immunotherapy. Theranostics. 2025;15(1):277–299. 39744236 10.7150/thno.101001 PMC11667223

[78] Tang J, He J, Guo H, Lin H, Li M, Yang T, Wang HY, Li D, Liu J, Li L, et al. PTBP2-mediated alternative splicing of IRF9 controls tumor-associated monocyte/macrophage chemotaxis and repolarization in neuroblastoma progression. Research. 2023;6:0033. 37040518 10.34133/research.0033 PMC10076020

