# Supplementary material for: Single-Cell RNA Sequencing Analysis Reveals the Role of Macrophage-Mediated CD44–AKT–CCL2 Pathways in Renal Tubule Injury during Calcium Oxalate Crystal Formation
Source: Research (Wash D C). 2025 May 6;8:0690. doi: 10.34133/research.0690 (PMC12053376; doi:10.34133/research.0690)
Supplement: Supplementary 1 — Figs. S1 to S16 Tables S1 to S5 [file research.0690.f1.zip › supplementary figures321.docx]

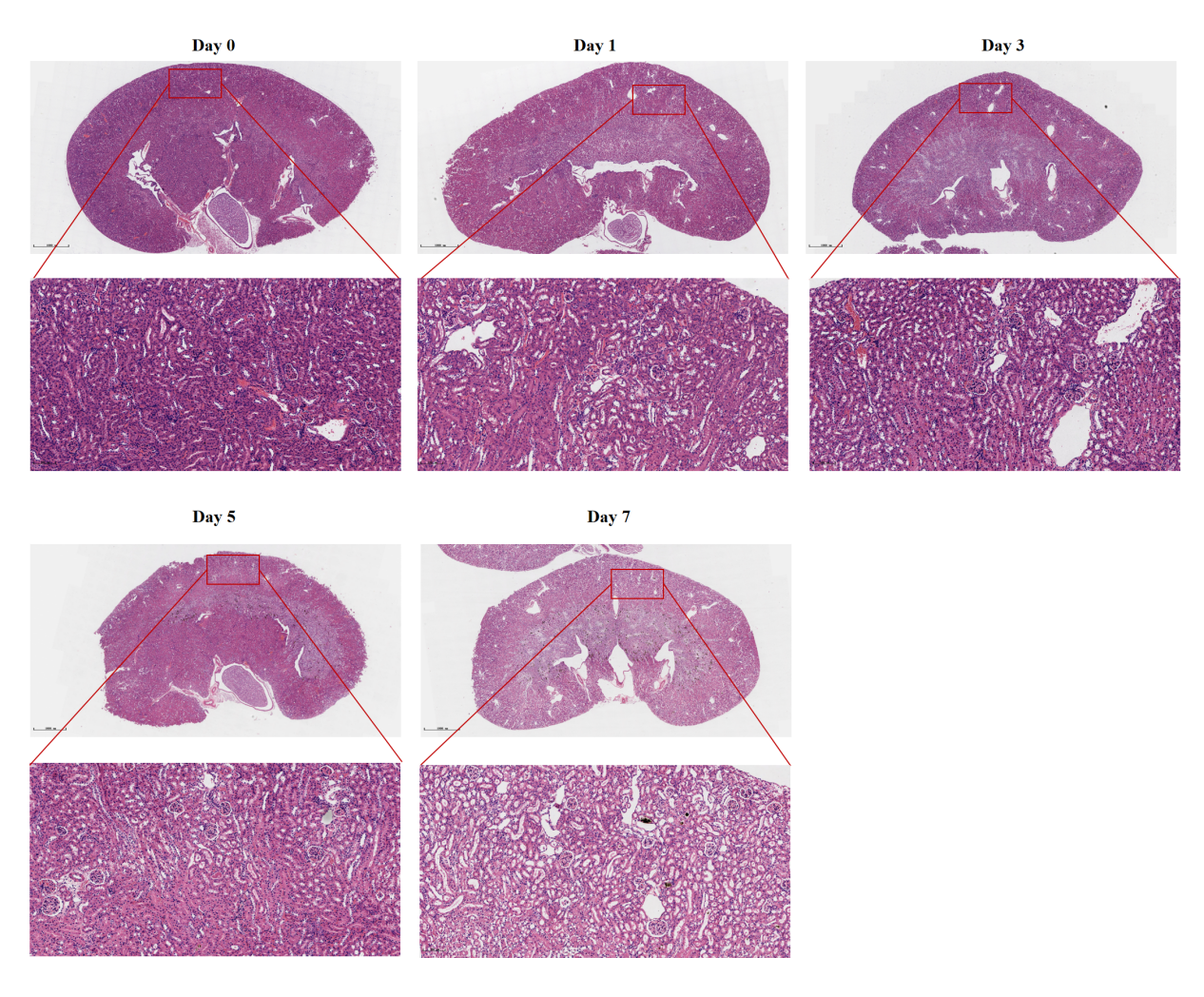


**Supplementary figure 1** Representative HE and von Kossa stainings of kidney sections of different days. Calcium oxalate depositions were black or brown dotplots.


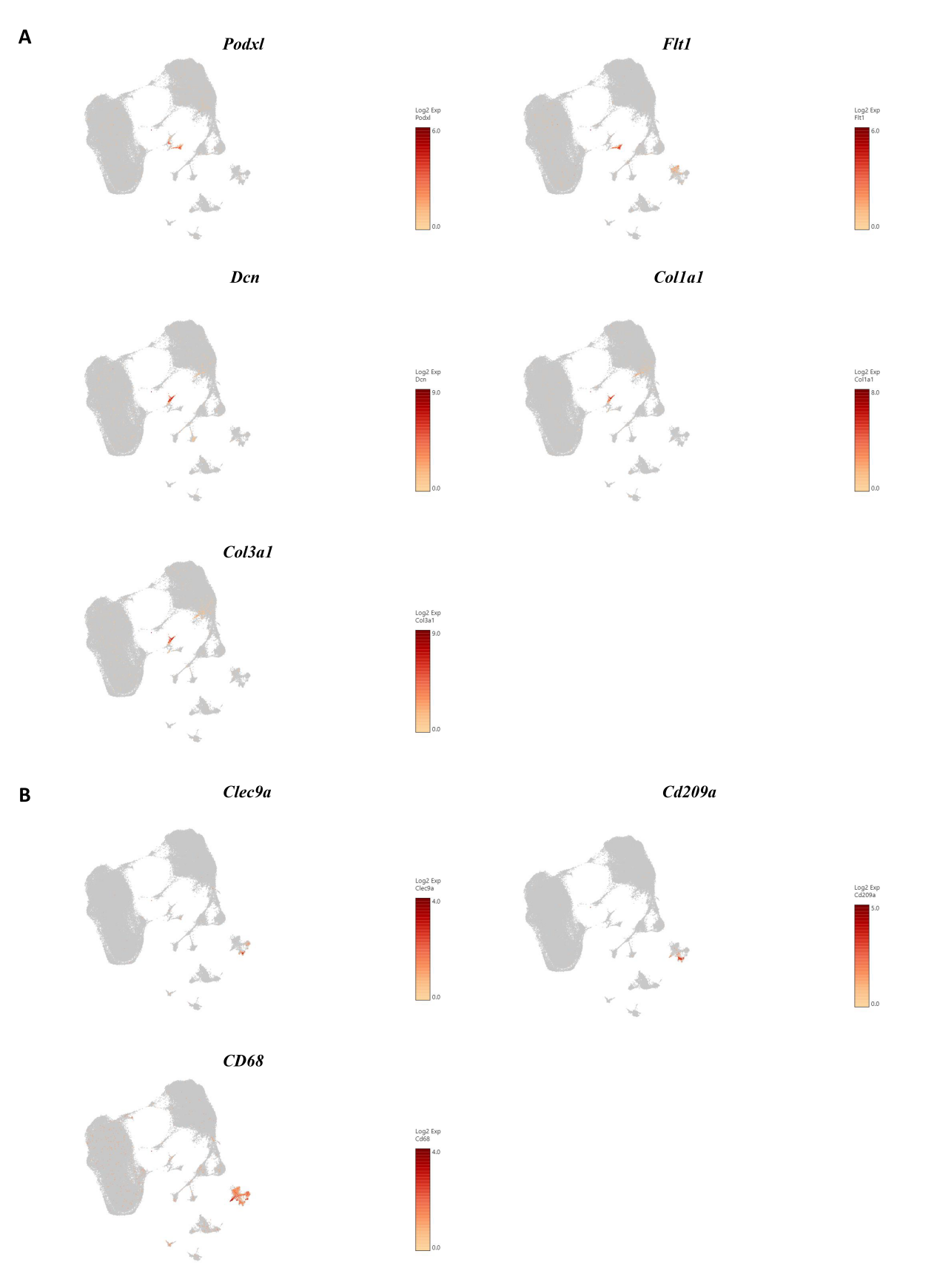


**Supplementary figure 2** Marker genes in UMAP plots. A) Marker genens of the mix cluster in UMAP plots. B) Marker genens of dendritic cell cluster in UMAP plots.


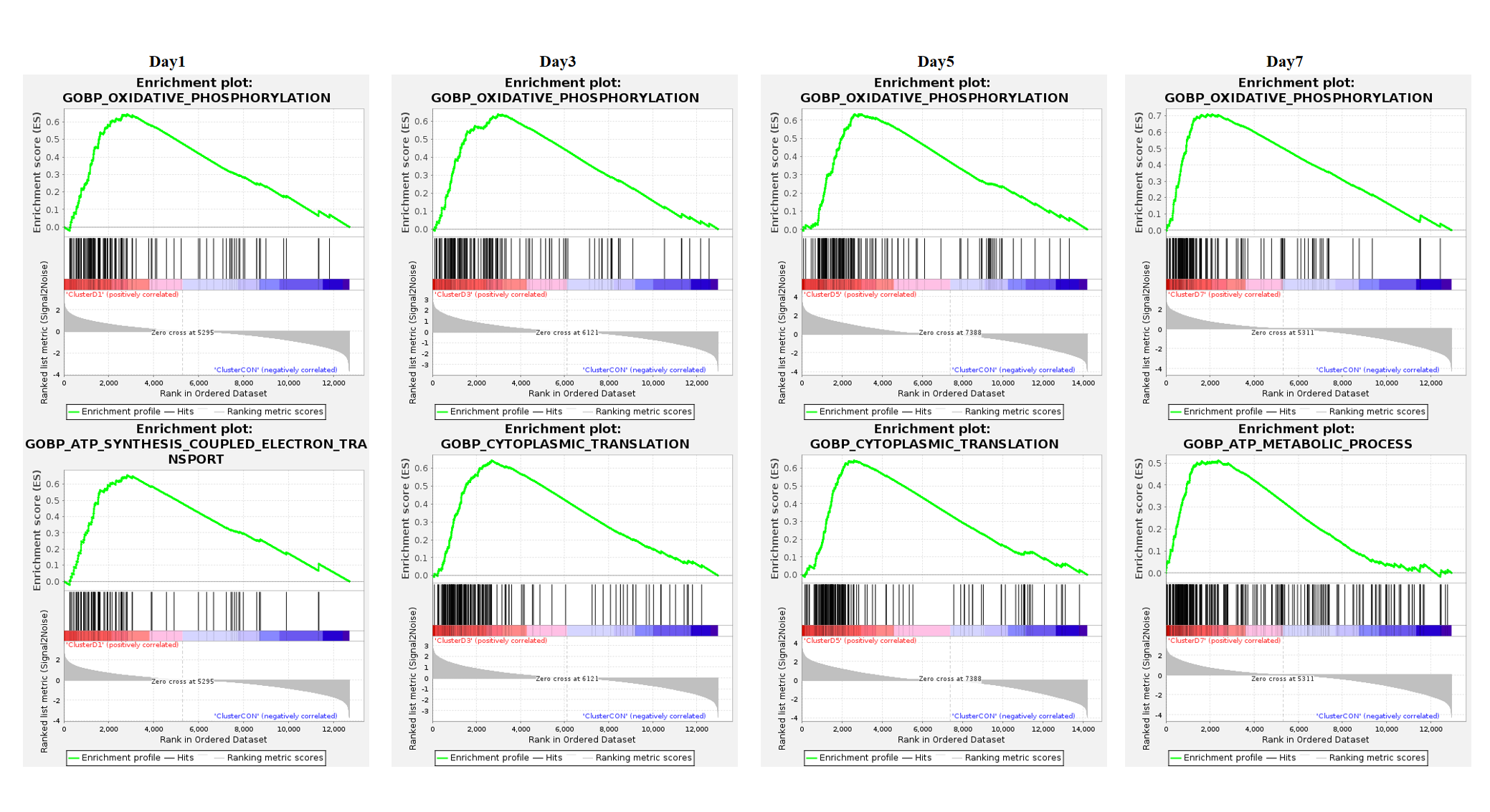


**Supplementary figure 3** GSEA analysis of proliferating PT cells.


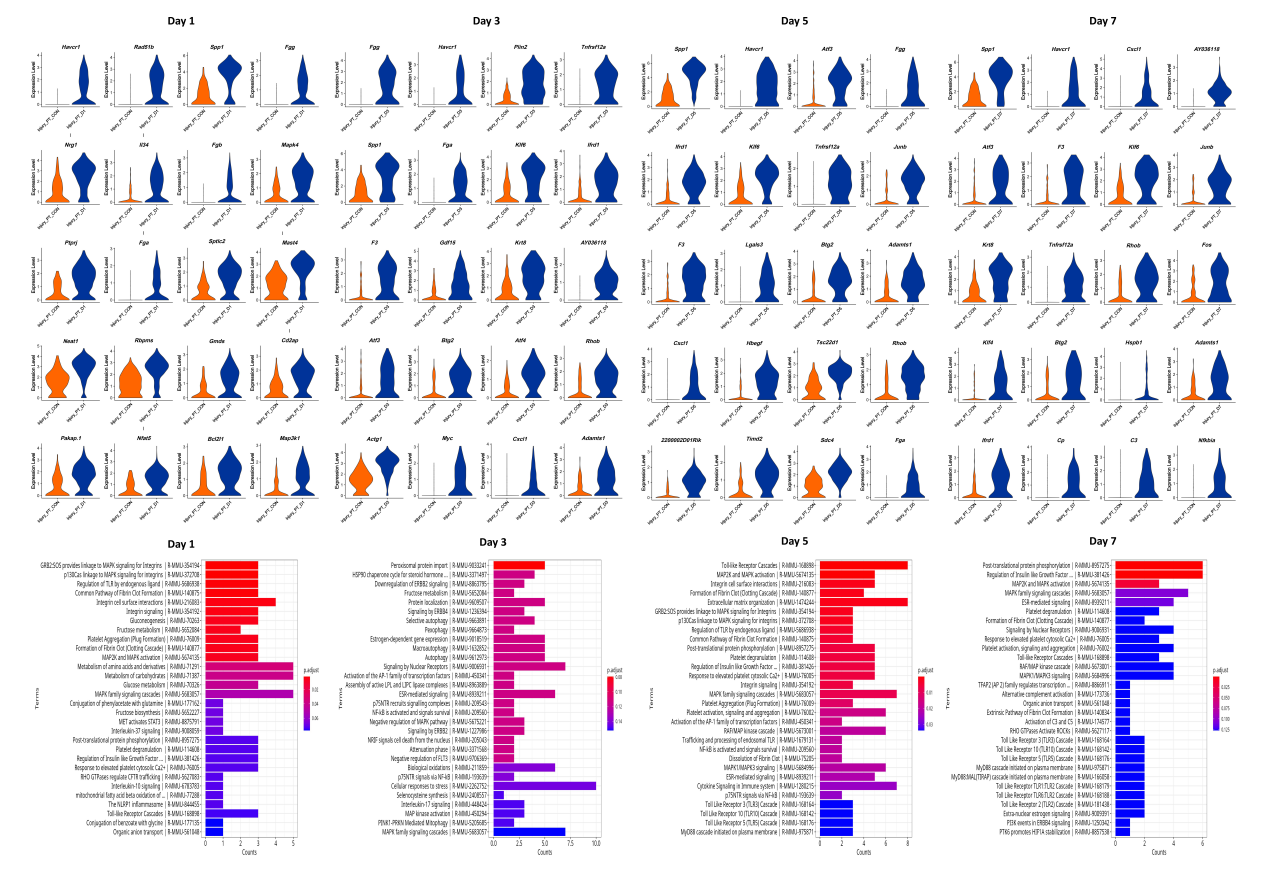


**Supplementary figure 4** Top genes and GO terms of injured PT cells.


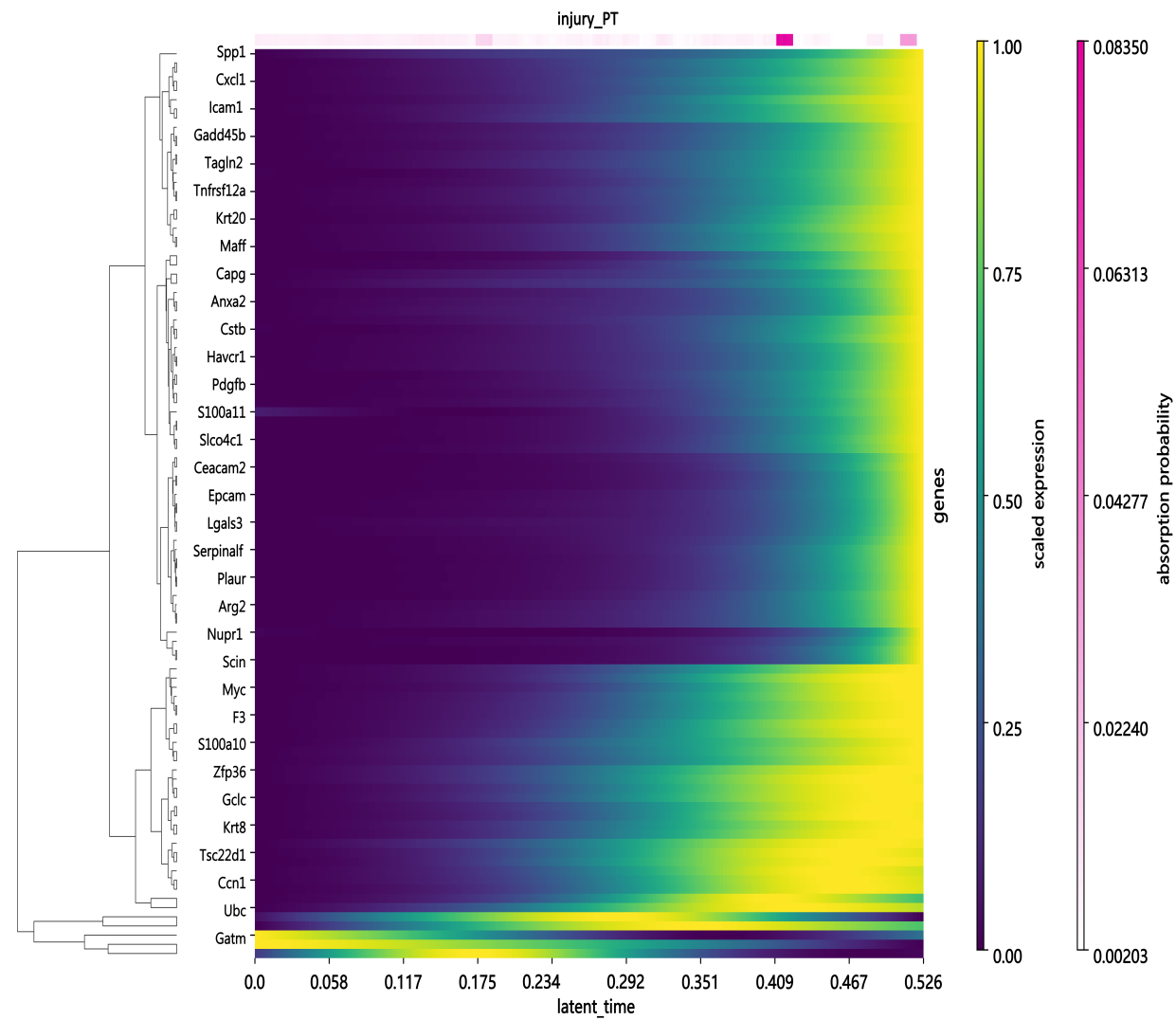


**Supplementary figure 5** Heatmap showing genes that significantly changed along the pseudotime in injury PTs clusters.


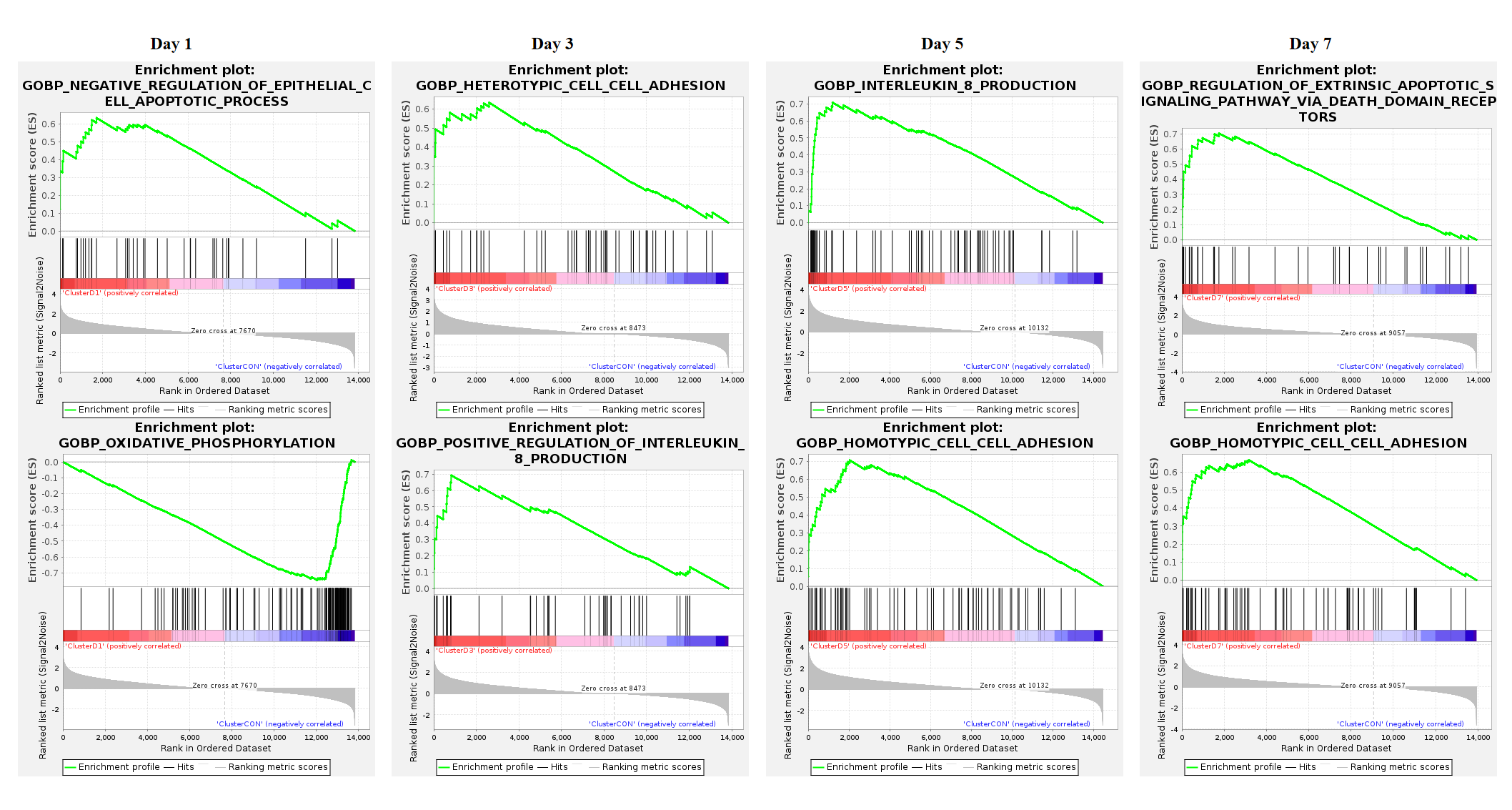


**Supplementary figure 6** GSEA analysis of injured PT cells.


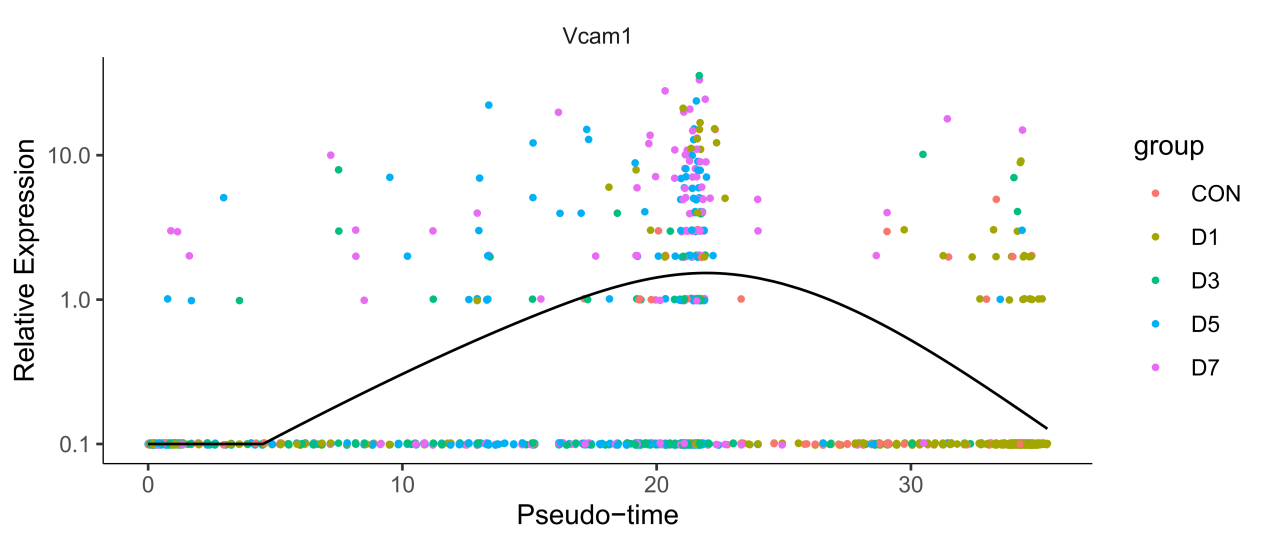


**Supplementary figure 7** Pseudotemporal expression locus of Vcam1among groups


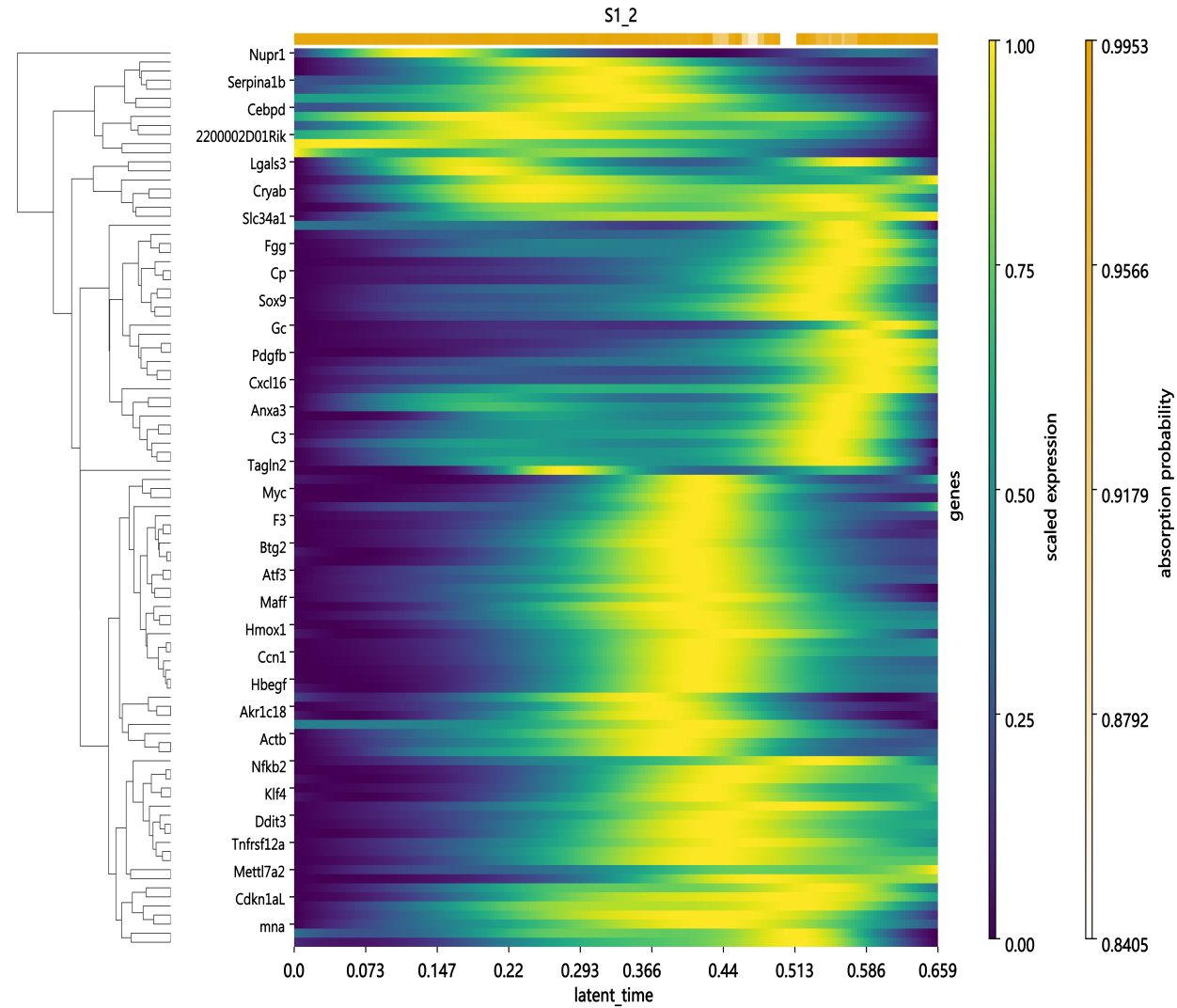


**Supplementary figure 8** Heatmap showing genes that significantly changed along the pseudotime in injury S1 clusters.


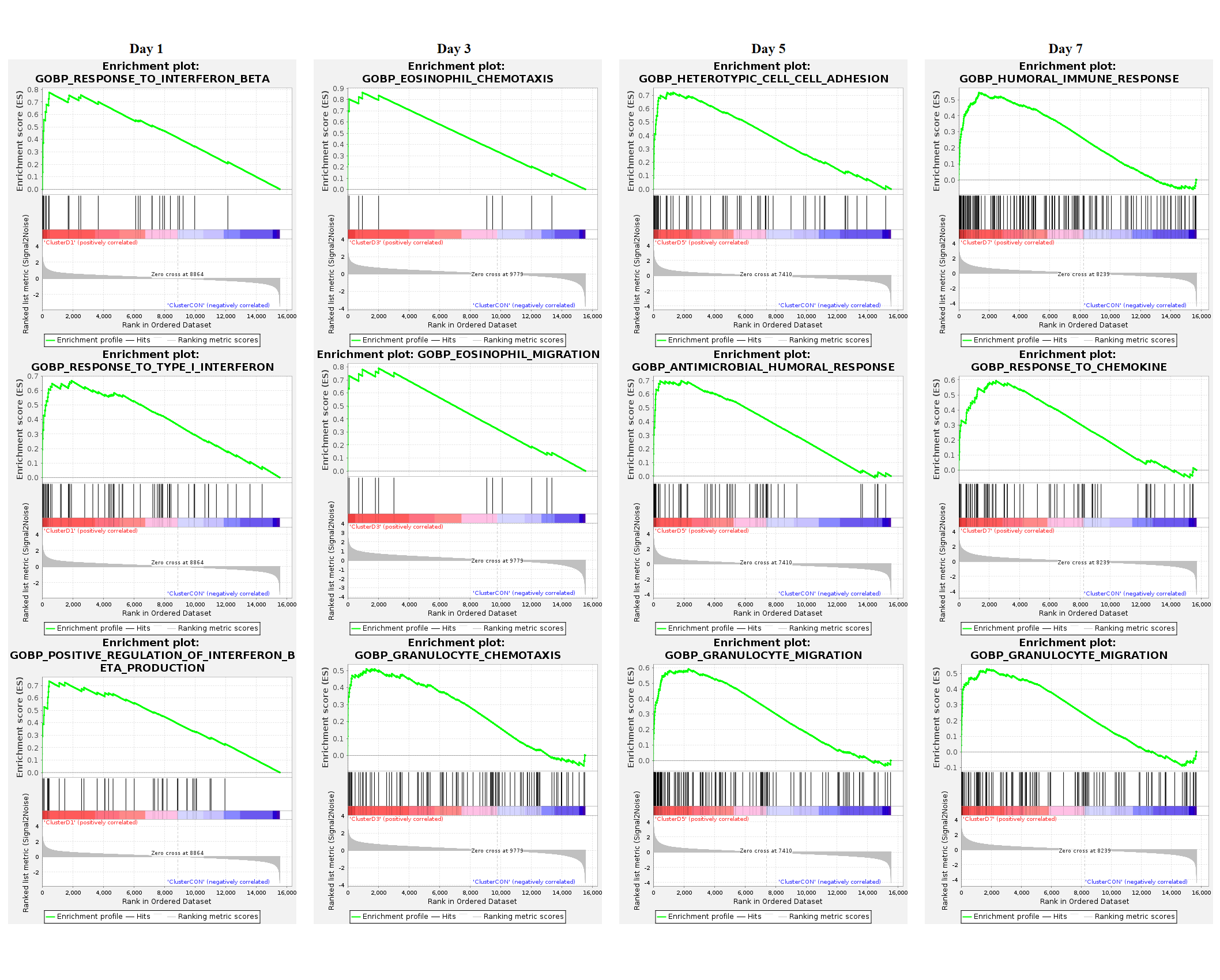


**Supplementary figure 9** GSEA analysis of S1 PT cells.


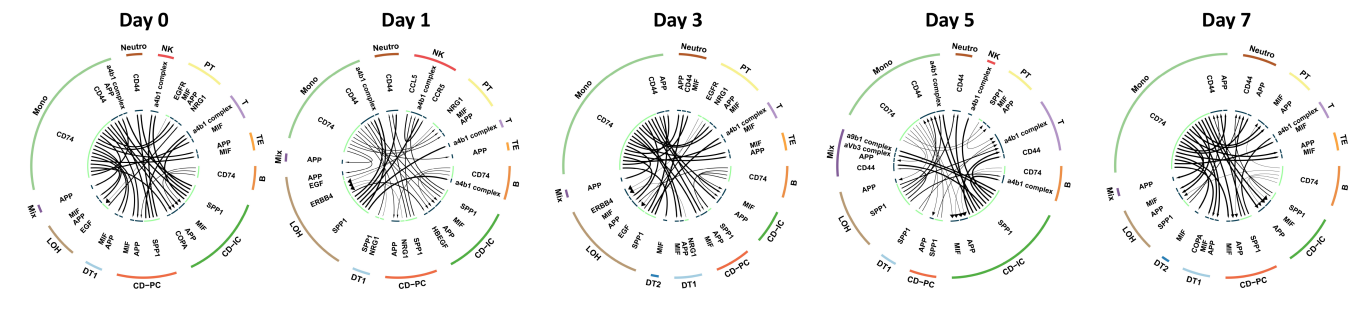


**Supplementary figure 10** The crosstalk between renal tubules and immune cells at different days.


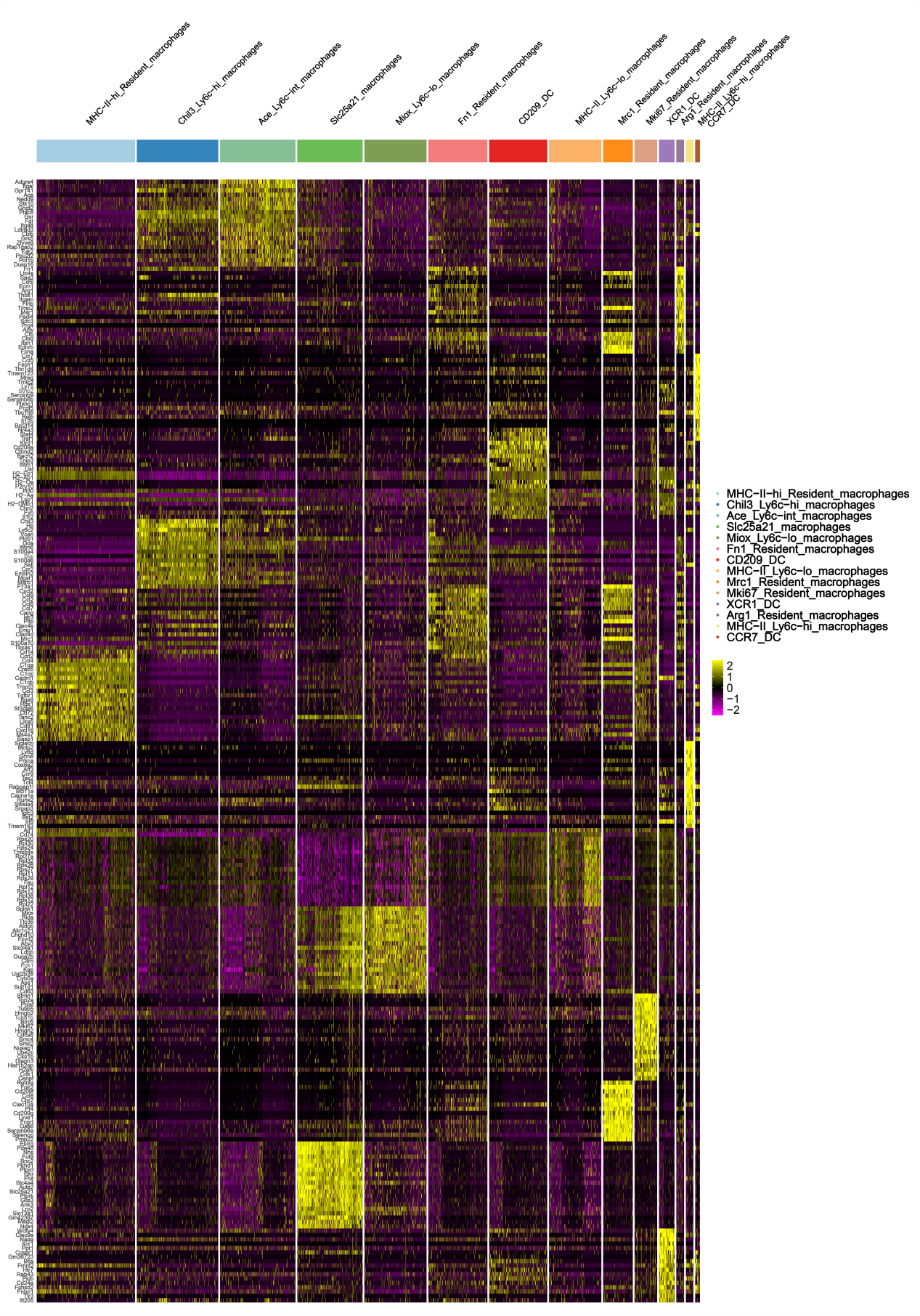


**Supplementary figure 11** Heatmap showing the expression of top marker genes across the monocyte clusters.


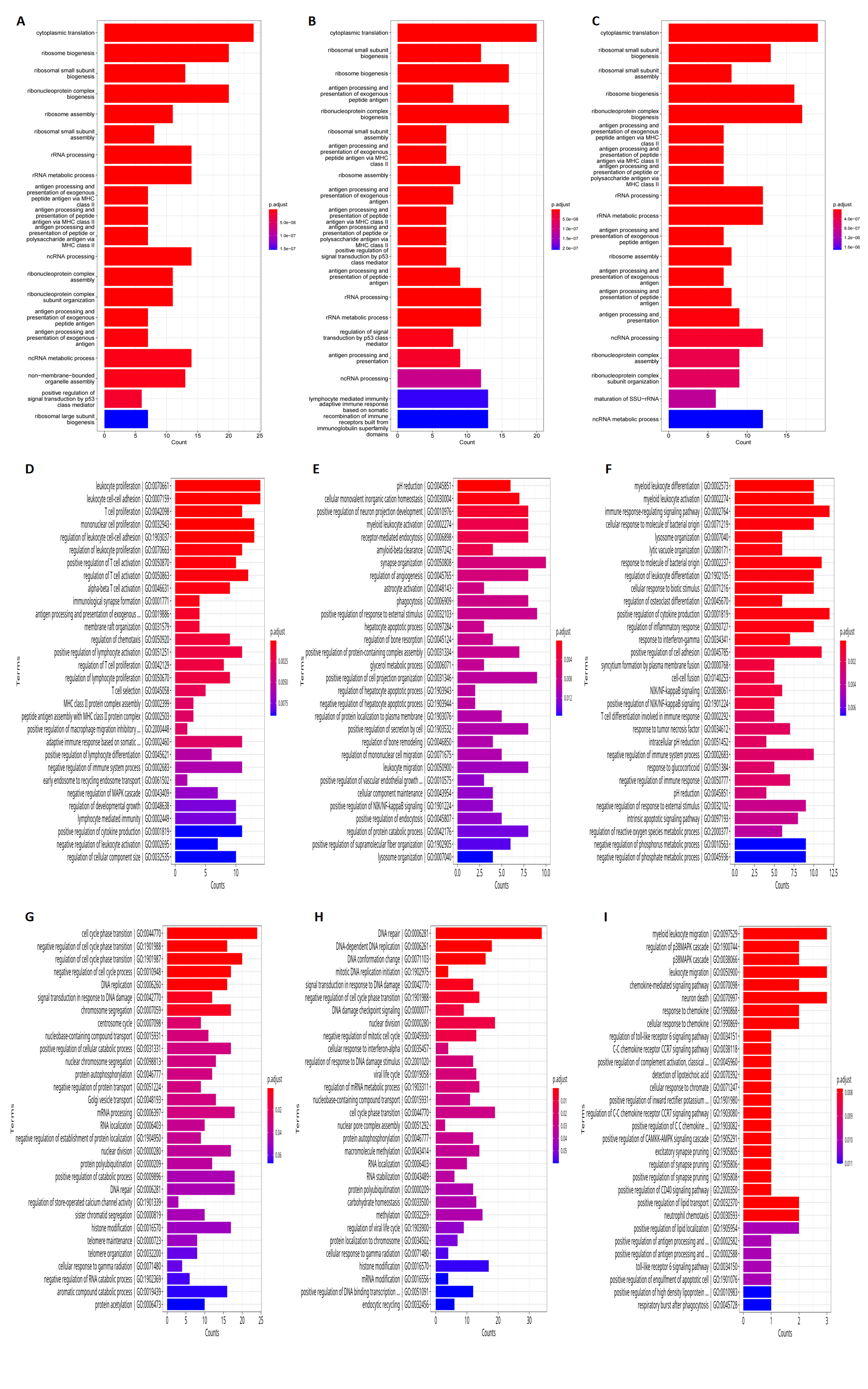


**Supplementary figure 12** GO terms of resident macrophages. A) GO terms of control Fn1 RMs. B) GO terms of control Mki67 RMs. C) GO terms of control MHC-II^hi^ RMs. D) GO terms of Fn1 RMs on day 1 of glyoxylate administration. E) GO terms of Fn1 RMs on day 3 of glyoxylate administration. F) GO terms of Fn1 RMs on day 7 of glyoxylate administration. G) GO terms of Mki67 RMs on day 5 of glyoxylate administration. H) GO terms of Mki67 RMs on day 7 of glyoxylate administration. I) GO terms of MHC-II^hi^  RMs on day 5 of glyoxylate administration.


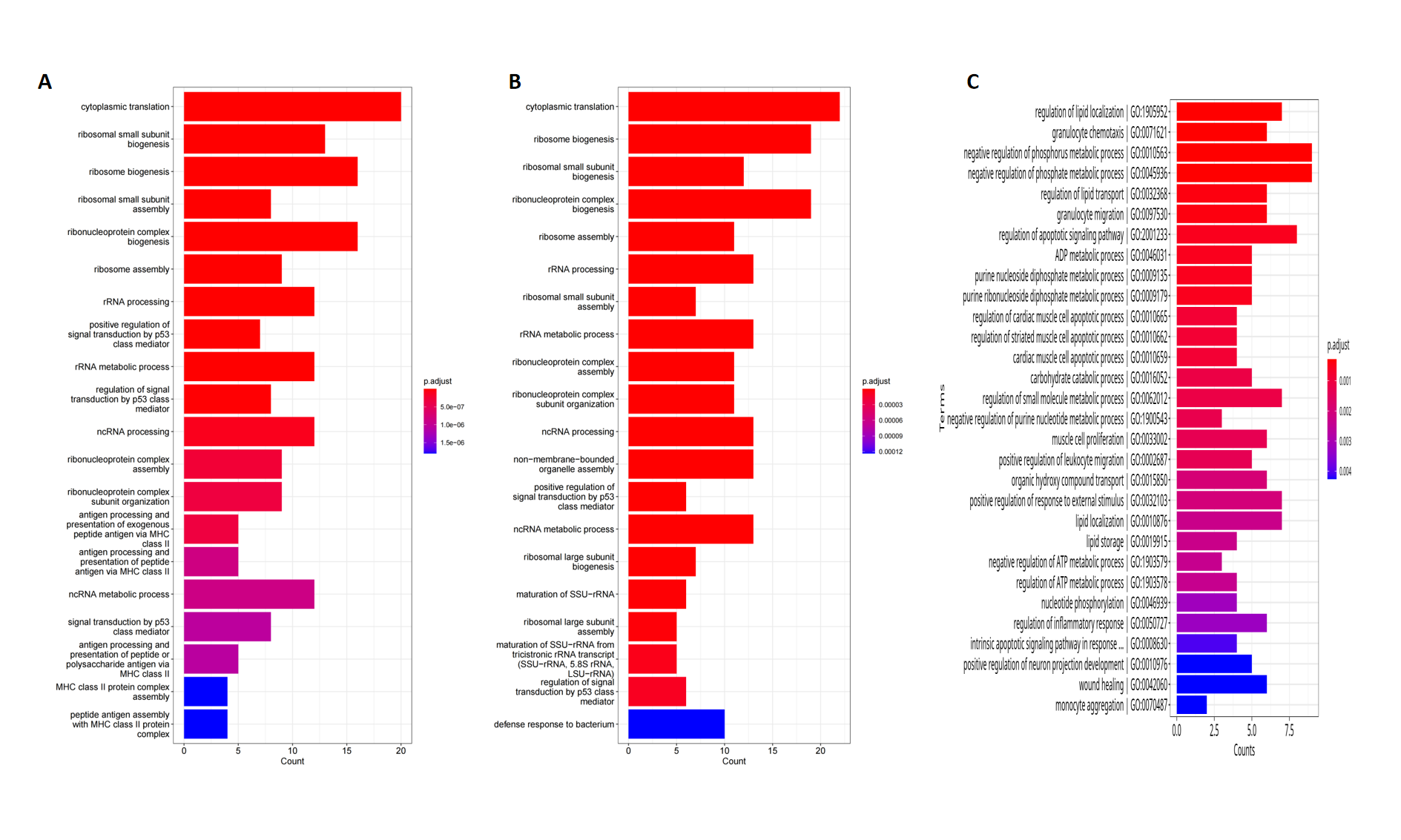


**Supplementary figure 13** GO terms of infiltrating macrophages. A) GO terms of control Miox Ly6c^lo^ macrophages. B) GO terms of control Chil3 Ly6c^hi^ macrophages. C) GO terms of Chil3 Ly6c^hi^ macrophages on day 5 of glyoxylate administration.


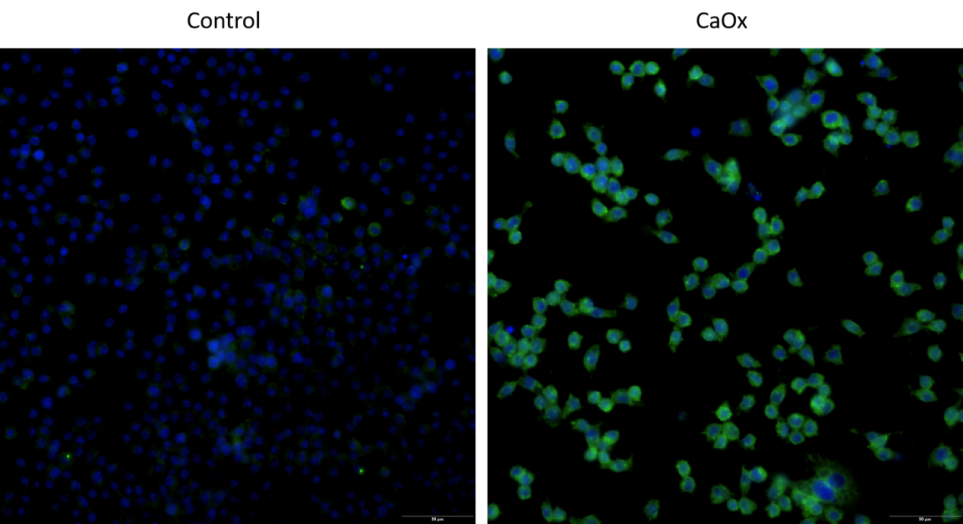

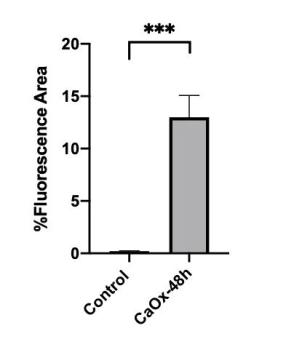


**Supplementary figure 14** Representative immunofluorescence of FN in macrophage stimulated with CaOx crystals.


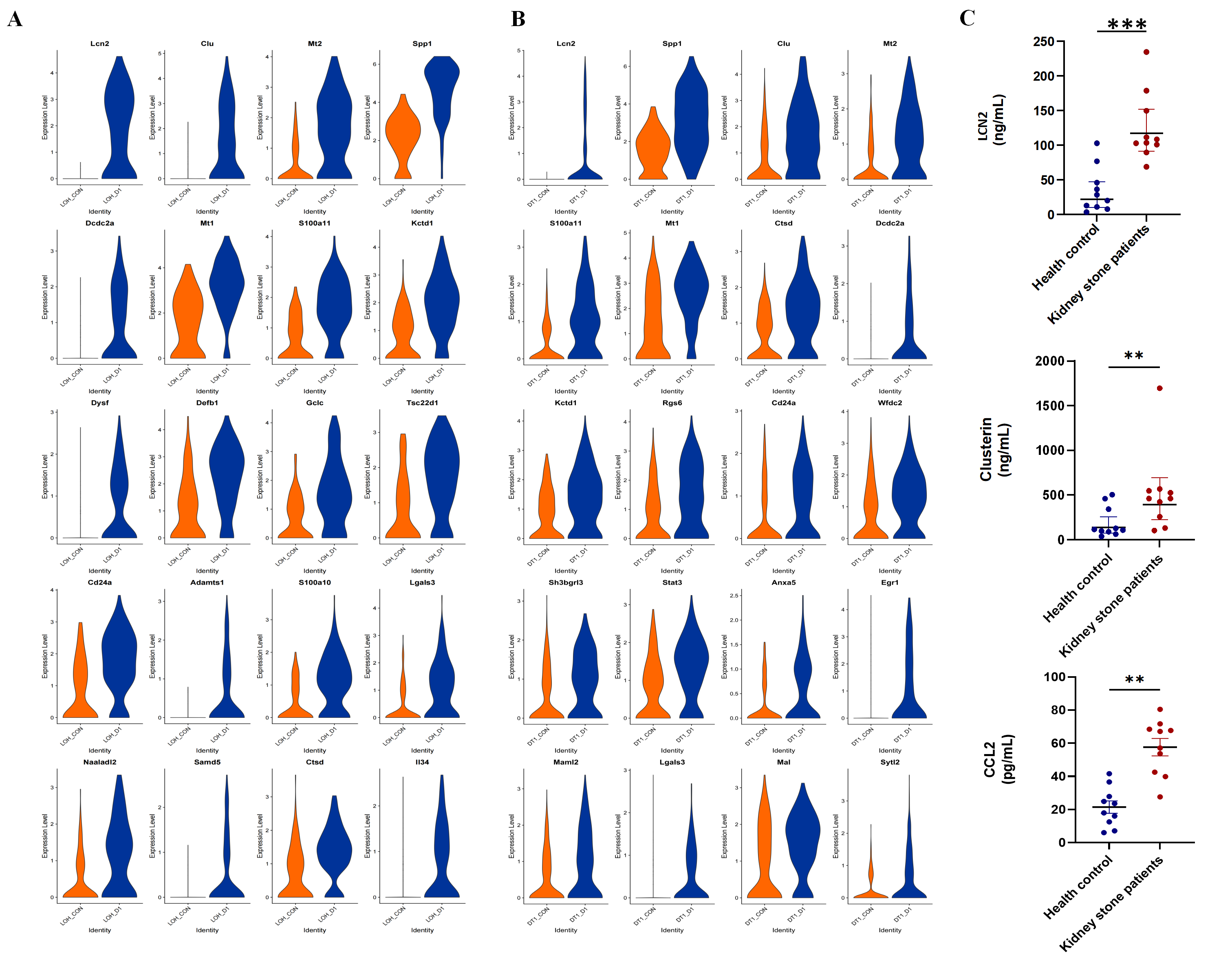


**Supplementary figure 15** Violin plots show the top genes expression in loop of Henle (A) and DT cluster on day 1 (B). ELISA results of LCN2, CLU and CCL2 protein levels in the urine of patients with CaOx stones and healthy controls (C). ** represented p < 0.01, ***represented p < 0.001.


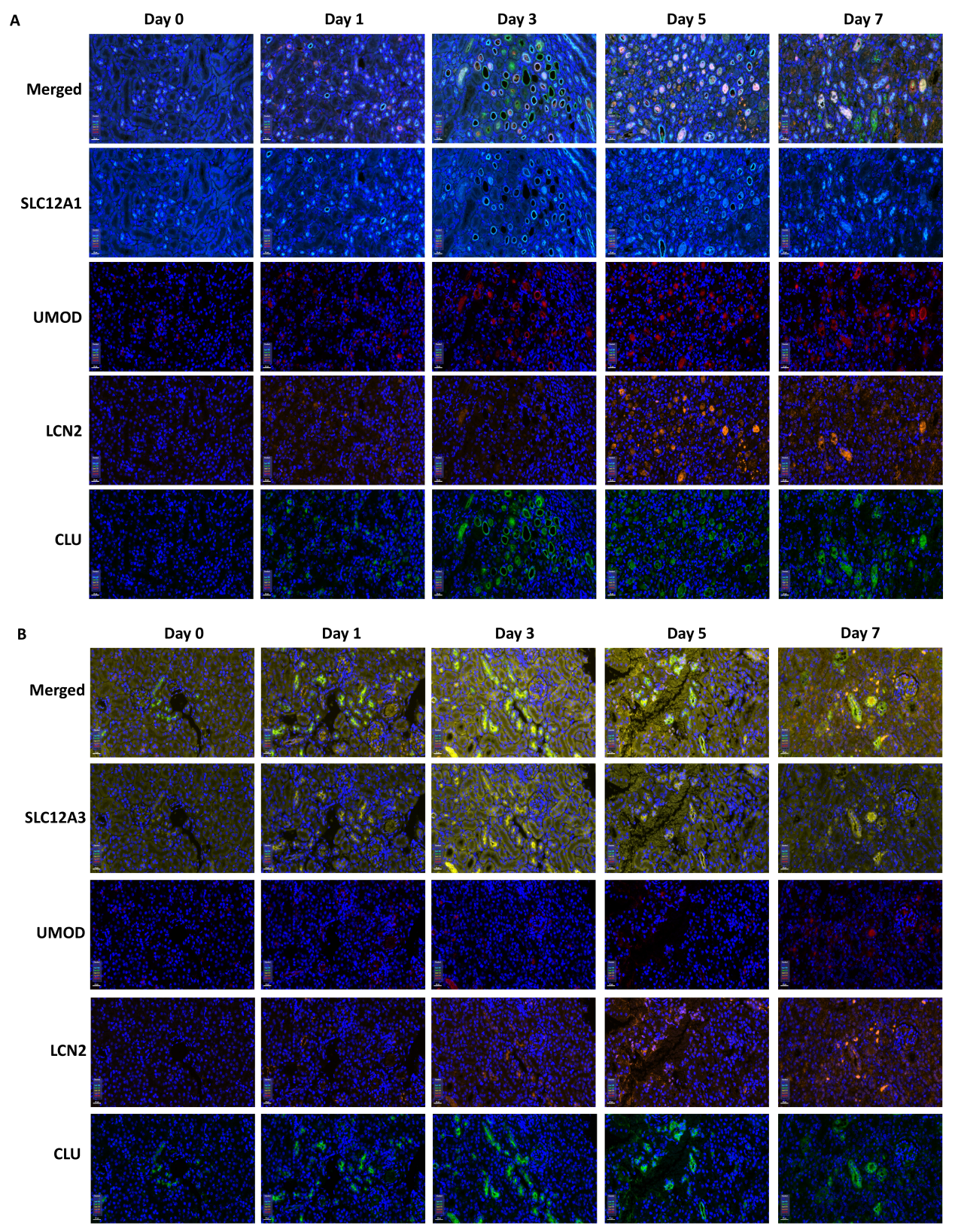


**Supplementary figure 16** Representative multiple fluorescent IHC of LCN2 and CLU in loop of Henle and distal tubules. (A) Representative multiple fluorescent of Slc12a1 (cyan), Umod (red), Lcn2(orange) and Clu (green) in loop of Henle. Slc12a3 and umod positive presented LOH. (B) Representative multiple fluorescent of Slc12a3 (yellow), Umod (red), Lcn2(orange) and Clu (green) in DT. Slc12a3 positive and umod negative presented the DT. DAPI (blue) for nuclei. Scale bar, 20 µm.
